# Population structure and adaptive differentiation in the sea cucumber *Apostichopus californicus* and implications for spatial resource management

**DOI:** 10.1371/journal.pone.0280500

**Published:** 2023-03-16

**Authors:** Natalie Lowell, Andy Suhrbier, Carolyn Tarpey, Samuel May, Henry Carson, Lorenz Hauser

**Affiliations:** 1 School of Aquatic and Fishery Sciences, University of Washington, Seattle, Washington, United States of America; 2 Pacific Shellfish Institute, Olympia, Washington, United States of America; 3 Washington Department of Fish and Wildlife, Olympia, Washington, United States of America; Universita degli Studi della Tuscia, ITALY

## Abstract

A growing body of evidence suggests that spatial population structure can develop in marine species despite large population sizes and high gene flow. Characterizing population structure is important for the effective management of exploited species, as it can be used to identify appropriate scales of management in fishery and aquaculture contexts. The California sea cucumber, *Apostichopus californicus*, is one such exploited species whose management could benefit from further characterization of population structure. Using restriction site-associated DNA (RAD) sequencing, we developed 2075 single nucleotide polymorphisms (SNPs) to quantify genetic structure over a broad section of the species’ range along the North American west coast and within the Salish Sea, a region supporting the Washington State *A*. *californicus* fishery and developing aquaculture production of the species. We found evidence for population structure (global fixation index (*F*_*ST*_) = 0.0068) with limited dispersal driving two patterns of differentiation: isolation-by-distance and a latitudinal gradient of differentiation. Notably, we found detectable population differences among collection sites within the Salish Sea (pairwise *F*_*ST*_ = 0.001–0.006). Using *F*_*ST*_ outlier detection and gene-environment association, we identified 10.2% of total SNPs as putatively adaptive. Environmental variables (e.g., temperature, salinity) from the sea surface were more correlated with genetic variation than those same variables measured near the benthos, suggesting that selection on pelagic larvae may drive adaptive differentiation to a greater degree than selection on adults. Our results were consistent with previous estimates of and patterns in population structure for this species in other extents of the range. Additionally, we found that patterns of neutral and adaptive differentiation co-varied, suggesting that adaptive barriers may limit dispersal. Our study provides guidance to decision-makers regarding the designation of management units for *A*. *californicus* and adds to the growing body of literature identifying genetic population differentiation in marine species despite large, nominally connected populations.

## Introduction

Over the last two decades, genetic studies have provided evidence that some marine species can develop spatial population structure despite very large population sizes and planktonic larval dispersal [1–3]. The traditional view of marine populations as demographically panmictic followed from the hypothesis that an apparent lack of barriers to dispersal in the marine environment and long-distance planktonic larval dispersal common to many marine species would result in high gene flow across large geographic regions [1]. However, it is likely that dispersal in marine populations is more restricted than previously thought, likely due to larval behavior [4, 5] and the presence of oceanographic barriers [6]. Moreover, selection can drive population differentiation in marine populations characterized by negligible genetic drift and/or high gene flow [7, 8]. Lastly, intrinsic reproductive barriers such as chromosome inversions or genetic incompatibilities may further restrict gene flow [9]. Characterization of genetic population structure is particularly important to effective management of exploited species. Genetic population structure can be used to define the scale of management actions to best meet management objectives [10, 11]. Ignoring spatial structure in fishery management may result in overexploitation of less productive or more accessible populations [12]. In turn, overexploitation may reduce population diversity supporting adaptability, and thus sustainability, for current and future uses [13, 14]. Additionally, demographic parameters such as the scale and direction of migration can be inferred from population structure [15], and this information can be used to inform sustainable management practices aimed at maintaining and rebuilding wild populations, such as those in marine protected areas [16].

Estimates of genetic population structure are also useful in informing best practices for aquaculture. Sourcing wild broodstock is a common practice in aquaculture production of species that are outplanted within their native range [17, 18]. If spatial population structure exists, then collecting wild broodstock from one distinct population and outplanting their seed into another can erode spatial population genetic structure and may lead to negative fitness consequences such as loss of local adaptations [19]. Conversely, sourcing broodstock and outplanting their seed within a single population could help maintain both the differentiation among wild populations and fitness of released offspring in the local environment if populations are locally adapted [20]. In recognition of such processes, the Department of Fisheries and Oceans Canada restricts translocation to within “shellfish transfer zones,” in part to minimize loss of population differentiation associated with aquaculture [21, 22].

The California sea cucumber, *Apostichopus californicus*, supports wild fisheries and is a novel aquaculture species [23], and thus would benefit from the characterization of spatial population structure. Demand for *A*. *californicus* has increased over the last several decades [24] as part of a global trend of growing demand for sea cucumbers [25]. Within Washington State, intense fishing pressure on this species peaked between 1988 and 1994, which reduced wild population sizes [25–27] and resulted in the closure of the Central Puget Sound fishery in 2014 [24]. Despite some management intervention, including reduced quotas and a closure during peak spawning season, stocks have been slow to recover [24]. Fishing pressure within Washington State is distributed differentially among six management areas [28], largely based on historic administrative boundaries and tribal fishing rights, but it is not known whether these areas represent biologically meaningful populations. Moreover, shellfish growers and scientists are developing methods for commercial aquaculture production of *A*. *californicus* [29] to supplement wild harvest, because demand remains high in overseas markets [24]. Defining management units relevant to fisheries would benefit the development of aquaculture as well, as it could help to develop guidelines for broodstock sourcing and stocking practices.

Population differentiation depends in part on life history. *A*. *californicus* reproduce through broadcast spawning, with females producing approximately 100,000–600,000 eggs per spawning event [30]. Larvae may remain in the planktonic stage for weeks to months until they settle, while adults are motile [31, 32]. Recent evidence suggests that adult sea cucumber dispersal can be facilitated through tumbling and floating behaviors, allowing them to take advantage of currents [33]. Formal estimates of life span are unavailable for *A*. *californicus*, but have been postulated to be about 12 years [34]. Other species of sea cucumbers live to be 5 to 10 years old [35]. Thus, dispersal potential is high in *A*. *californicus* because all life history stages are motile and *A*. *californicus* are potentially long-lived, which could lead to high population connectivity. The life history strategy of *A*. *californicus* is that of a periodic strategist with long generation time, moderate reproductive effort, high batch fecundity and low investment per offspring [36]. This strategy is an adaptation to situations where environmental variation affecting juvenile survival is unpredictable but occurs on a large geographic scale [37], which in turn may result in high interannual variation in recruitment [36], high variance in reproductive success and low *N*_*e*_/*N* ratios [38]. The life history of *A*. *californicus* may therefore suggest higher population differentiation than expected from census population sizes and high susceptibility to overfishing [36].

*A*. *californicus* is distributed from Baja California, Mexico, to Alaska, from the lower intertidal to depths of 250 *m* [31, 39], a region containing oceanographic barriers to dispersal that could shape population connectivity in *A*. *californicus*. For example, the Salish Sea, a large estuary divided into sub-basins by sills, is known to harbor genetically distinct populations in Pacific Cod, *Gadus macrocephalus* [40], and Brown Rockfish, *Sebastes auriculatus* [41]. The Victoria Sill and Admiralty Inlet are two potentially important oceanographic barriers to dispersal within the Salish Sea. The Victoria Sill is the outermost oceanographic barrier dividing the Salish Sea from the Strait of Juan de Fuca, which connects the Salish Sea to the outer coast [42]. The two sills at Admiralty Inlet separate the main Puget Sound basin from the San Juan Islands region and the Georgia Strait [43]. Another potential oceanographic barrier is the North Pacific Current (NPC), which bifurcates as it approaches the Pacific coast of North America into the northward Alaska and southward California currents. The NPC was already identified as an oceanographic barrier to dispersal in *A*. *californicus* [22] and the Bat Star, *Patiria miniata* [44].

Spatial population structure correlated with oceanographic processes was recently characterized in *A*. *californicus* in the region near British Columbia, Canada [22, 45]. These authors found evidence for local adaptation associated with mean bottom temperature, and to a lesser extent, surface salinity and bottom current velocity [45]. However, this study was restricted to British Columbia, and did not include the southern extent of the range, in particular the southern Salish Sea, where the majority of sea cucumbers are harvested in the contiguous United States [46] and where the fishery has experienced closures due to overfishing [24]. Thus, estimates of spatial population structure are particularly needed in this region. Sampling the northern and southern extents of the region, beyond British Columbia, is also needed, as fisheries for *A*. *californicus* extend from Alaska to California.

In this study, we quantified and described patterns of population differentiation for *A*. *californicus* to inform effective management of wild populations and facilitate the development of management guidelines for aquaculture. We sampled populations at small and large geographic scales to test for both fine- and broad-scale population structure, particularly in regions that allow us to test hypotheses of potential oceanographic barriers to dispersal. Using restriction site-associated DNA (RAD) sequencing, we quantified genetic structure and investigated its potential drivers. Specifically, we tested whether limited dispersal and local adaptation shape population structure. We compared our results with existing estimates of spatial population structure to identify common patterns and build hypotheses about drivers of differentiation in the species. To contextualize our results for managers, we developed a two-population model to evaluate which conditions of effective population size, migration rate, number of generations of drift resulted in pairwise population differentiation within the range observed in this study.

## Materials and methods

### Sample collection

**Fig 1:**
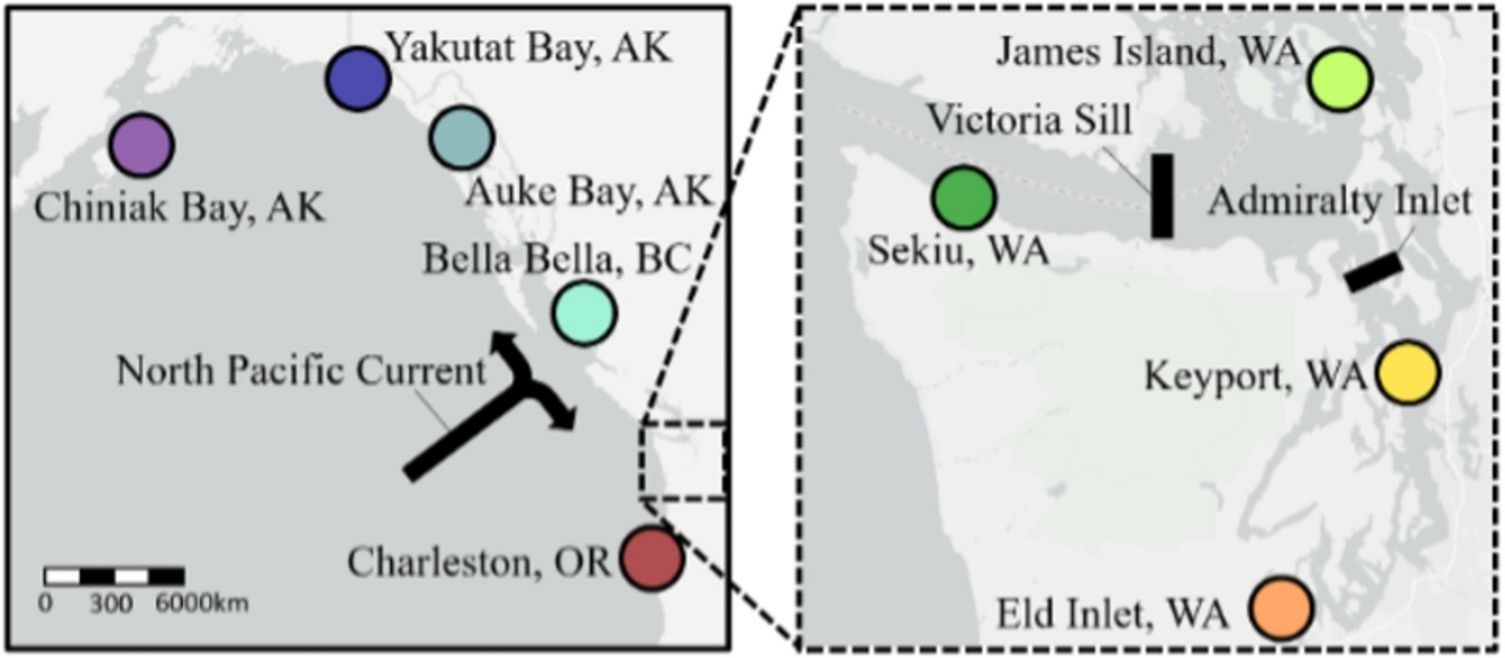
A map of collection sites. Collection sites are located in the Northeast Pacific Ocean, including an inset of the southern Salish Sea (right) and potential oceanographic barriers investigated in this study.

Adult *A*. *californicus* were collected by scuba divers from nine collection sites along the Pacific Coast of North America, ranging from Alaska to Oregon, including four collection sites within the southern Salish Sea (Table 1, Fig 1). Sample size ranges from 12 to 53 samples per collection site. For each animal, a tissue sample was excised from a radial muscle band and stored in 100% ethanol.

**Table 1:** General information by collection site.

*Site*, *State*	*SS*	*PS*	*NPC*	*Code*	*Lat*	*Long*	*I*_*S*_	*I*_*R*_	*H*_*E*_	*H*_*O*_	*F*_*IS*_	*S*_*P*_
Chiniak Bay, AK	O	O	N	CB_AK	57.712	-152.357	27	19	0.278	0.251	0.073	0.931
Yakutat Bay, AK	O	O	N	YB_AK	59.716	-139.846	41	39	0.279	0.250	0.091	0.976
Auke Bay, AK	O	O	N	AB_AK	58.367	-134.668	12	10	0.275	0.240	0.093	0.839
Bella Bella, BC	O	O	N	BB_BC	52.152	-128.139	41	32	0.286	0.232	0.120	0.950
Sekiu, WA	O	O	S	SK_WA	48.160	-124.175	47	47	0.279	0.234	0.147	0.980
James Island, WA	I	O	S	JI_WA	48.513	-122.774	53	50	0.277	0.233	0.151	0.971
Keyport, WA	I	I	S	KP_WA	47.701	-122.634	44	42	0.278	0.233	0.157	0.966
Eld Inlet, WA	I	I	S	EI_WA	47.147	-122.935	53	48	0.280	0.234	0.154	0.973
Charleston, OR	O	O	S	CH_OR	43.340	-124.378	39	38	0.277	0.233	0.155	0.967

Column Site, State refers to the collection site name, followed by the state or province using two letter codes, SS refers to whether inside (I) or outside (O) the Salish Sea, NPC refers to whether north (N) or south (S) of the North Pacific Current (NPC), Code contains the collection site code used in tables and figures, Lat and Long refer to latitude and longitude of collection sites, I_S_ refers to number of individuals sequenced (excluding replicates), I_R_ refers to number of individuals retained in analyses (excluding replicates), H_E_ refers to mean expected heterozygosity, H_O_ refers to mean observed heterozygosity, F_IS_ refers to mean locus F_IS,_ and S_P_ refers to the proportion of SNPs that are polymorphic.

### DNA library preparation

DNA was extracted from tissue samples using the EZNA Mollusc DNA Kit (OMEGA Bio-tek, Norcross, GA, USA) and the Qiagen DNeasy Kit (Qiagen, Germantown, MD, USA). DNA was quantified using the Quant-iT PicoGreen dsDNA Assay Kit (Thermo Fisher Scientific, Waltham, MA, USA) and DNA quality was checked by gel electrophoresis. DNA concentration was normalized to 500 *ng* in 20 *μL* of PCR-grade water. We selected samples with high DNA quality for restriction site associated DNA (RAD) sequencing and RAD libraries were prepared following standard protocols [47]. Briefly, DNA samples were barcoded with an individual six-base identifier sequence attached to an Illumina P1 adapter. Samples were then pooled into sub-libraries, containing approximately 12 individuals. Sub-libraries were sheared using a Bioruptor sonicator and size selected to 200–400 *bp* using a MinElute Gel Extraction Kit (Qiagen, Germantown, MD, USA). P2 adapters were ligated to DNA in sub-libraries and amplified with PCR using 12–18 cycles as in Etter et al. [47]. Finally, amplified sub-libraries were combined into pools of approximately 72 individuals. Paired-end 2 x 150-base pair sequencing was performed on an Illumina HiSeq4000 (San Diego, California, USA) at the Beijing Genomics Institute and the University of Oregon Genomics and Cell Characterization Core Facility. Only forward reads were used for analysis. To estimate genotyping error, 14 individuals were sequenced twice.

### Genotyping individuals

Raw RAD sequencing data were demultiplexed and sequences were trimmed to 140 *bp* using the *process_radtags* module in the pipeline *STACKS* v.1.44 [48]. A threshold of 800,000 reads was used to exclude poorly sequenced individuals. Because a genome was not available for *A*. *californicus*, we aligned individual sequences to the genome of a closely related species, *A*. *parvimensis* (GenBank accession number = GCA_000934455.1). The *A*. *parvimensis* genome was 760,654,621 *bp*, with 21,559 scaffolds and an N50 size of 9,587. We retained reads with a minimum mapping quality score of 20. Then, we used *dDocent* v.2.7.8 to perform a reference-guided locus assembly using the filtered reads and default parameters [49]. Additionally, a parallel *de novo* assembly was performed, which produced nearly identical results for population structure (Table A and Fig A in S1 File) and 1.8–2.8% lower mean expected heterozygosity, 0.9–1.8% higher mean observed heterozygosity, and 1.2–3.3% higher proportions of polymorphic SNPs (Table B in S1 File) than in the with-reference assembly, although with similar patterns across collection sites. The reference-guided assembly was retained for further analyses due to decreased confidence in identifying genotyping errors in the *de novo* assembly [50].

We used *vcftools* v.0.1.16 [51] to remove indels and to retain only single nucleotide polymorphisms (SNPs) with a minimum quality score of 20, minimum minor allele frequency of 0.05, and maximum missing data per locus of 30% across collection sites. Individuals with more than 30% missing data across SNPs were removed. In cases of multiple SNPs per RAD tag, we retained the SNP with the highest minor allele frequency [52]. SNPs that were not in Hardy Weinberg Equilibrium (HWE) were considered sequencing errors or poorly assembled loci and were removed from our data set, as selection and inbreeding are unlikely to cause significant deviations from HWE equilibrium at biallelic loci [53]. We tested SNPs for deviations from HWE using the R package *genepop* v.1.1.4 [54]. SNPs were identified as being out of HWE if they had a *q*-value below 0.05 in at least 2 of the collection sites after correcting for false discovery rate, following Waples [53].

### Population genetic structure analyses

We used a suite of R packages, stand-alone software, and custom scripts in the programming language R v.3.5.0 [53, 55] to quantify genetic diversity and population structure. Mean expected heterozygosity, observed heterozygosity, and the inbreeding coefficient (*F*_*IS*_) per SNP were calculated using the R package *genepop*. The proportion of polymorphic SNPs per collection site was calculated using a custom R script.

To investigate population structure, we first calculated Weir-Cockerham fixation index (*F*_*ST*_) [56] to quantify population differentiation using the R packages *genepop* and *hierfstat* v.0.5.7. Exact *G*-tests [57] were used to test for significant genic differentiation using the R package *genepop*. To investigate patterns of spatial differentiation among collection sites, the R package *adegenet* v.2.1.1 [58] was used to conduct discriminant analyses of principal components (DAPC), a multivariate method that summarizes the between-group variation (i.e., population structure), while minimizing within-group variation [59]. The built-in optimization algorithm was used to retain the number of principal components that minimized over-fitting and under-fitting of the model. To determine the potential number of underlying populations, the program *ADMIXTURE* v.1.3.0 was used to conduct a clustering analysis [60]. Specifically, *ADMIXTURE* uses a maximum likelihood-based approach to estimate individual ancestries across different assumed numbers of populations, with the best fit selected using cross-validation. To examine the presence of hierarchical population structure, we conducted analyses of molecular variance (AMOVA) using the *ade4* method of the R package *poppr* v.2.8.1 [61]. Significance of AMOVAs was determined using permutation tests with 1,000 iterations. Using AMOVA, we investigated whether the following oceanographic barriers limit dispersal: 1) the Victoria Sill (Victoria Sill grouping), 2) Admiralty Inlet (Admiralty Inlet grouping), and 3) the North Pacific Current (NPC grouping). Because AMOVAs for each oceanographic barrier include sites in an area with other potential oceanographic barriers, we added a fourth grouping of all three oceanographic barriers (All Barriers grouping) to investigate the relative role of oceanographic barriers compared to other factors. Additionally, we conducted an AMOVA by state or province (State grouping). Although not biologically meaningful, we included the State grouping to determine how much genetic variation is captured by regional management boundaries. Isolation-by-distance (IBD) was tested with Mantel tests [62] in R as correlation between linearized *F*_*ST*_ [63] using all SNPs and shortest Euclidean distance through water (in-water distance hereafter) approximated in Google Maps [64], between all pairs of collection sites. Following Xuereb et al. [65], we also tested IBD in the northern and southern population section separately. Following Buonaccorsi et al. [41], we estimated mean dispersal distance from the slope of the regression of linearized *F*_*ST*_ and in-water distance. We used this one-dimensional model because it is an appropriate approximation for coastal species with dispersal distances greater than the second dimension of the habitat [66] and because spatial patterns do not seem to affect empirical IBD patterns [67]. We estimated mean dispersal distance from a set of potential population density estimates as population density estimates were unavailable.

### Identifying putative adaptive differentiation

We used two approaches to investigate putatively adaptive SNPs: *F*_*ST*_ outlier detection and gene-environment association. *F*_*ST*_ outlier detection is used to identify loci potentially under spatial selection [68, 69], although this method does not identify the potential cause of selection. Although gene-environment association does not explicitly test whether such associations are adaptive, this method is used to identify locus-environment associations as evidence for potential local adaptation [70]. SNPs were classified as putatively adaptive if they were detected as *F*_*ST*_ outliers or if they were significantly correlated to environmental predictors using gene-environment association.

For *F*_*ST*_ outlier detection, two methods were used: the program *Bayescan* v.2.1 [68] and the R package *OutFLANK* v.0.2 [69]. *Bayescan* first applies linear regression to decompose *F*_*ST*_ into a population- and a locus-specific component. Using these components as Bayesian priors, the program estimates the posterior probability that a locus is under selection [68]. *OutFLANK* detects *F*_*ST*_ outliers using a maximum likelihood approach. The program first infers a distribution of neutral *F*_*ST*_ from a trimmed distribution of empirically collected *F*_*ST*_ values and uses this neutral distribution to identify outliers. *OutFLANK* advances earlier *F*_*ST*_ outlier methods [71] by accounting for sampling error and non-independent sampling of populations, and has lower false positive rates compared to other *F*_*ST*_ outlier methods [69]. We used default parameters in both programs, including a false discovery rate of 0.05. SNPs were classified as *F*_*ST*_ outliers if they were detected with either program, to include SNPs under weak selection, which are likely the majority of SNPs under selection [72].

Prior to investigating gene-environment association, we gathered estimates for oceanographic variables at each collection site using the Bio-Oracle and Bio-Oracle 2 databases [73], which contain geophysical, biotic, and environmental data layers for marine realms, through the R package *sdmpredictors* v.0.2.8 [74]. We selected a broad suite of 29 oceanographic variables (including temperature, current velocity, salinity, and pH; complete list in Table C in S1 File), including those used by Xuereb et al. [45] for comparison. Where possible, oceanographic variables for both sea surface and mean bottom depth (near-bottom) were used to account for the conditions experienced by pelagic larvae and benthic adults respectively. The Eld Inlet, Washington collection site was removed from these analyses because environmental predictor data were not available. We also calculated Pearson’s correlation coefficients among predictor variables (Table D in S1 File) using the R package *psych* [75] and interpreted results in light of these correlations.

To investigate genomic evidence for local adaptation, gene-environment associations were explored using a univariate association method, *Bayenv2* [70], and a multivariate method, redundancy analysis (RDA). We used both methods as each has an advantage: the interpretation of results for univariate methods like *Bayenv2* can be clearer than multivariate methods for gene-environment association, and multivariate methods such as RDA have lower false positive rates and greater sensitivity for detecting weak and multi-locus selection [75].

The program *Bayenv2* [76] uses a univariate Bayesian framework to test for significant correlation between allele frequencies and environmental predictor variables, accounting for population structure by first estimating covariance among loci. Correlations with a minimum Bayes Factor of 10, or minimum “strong” support [77], were retained in the analysis.

Redundancy analyses were performed in the R package *vegan* v.2.5–6 [78]. Redundancy analysis summarizes the variation in a set of response variables (here, the allele frequencies) due to a set of explanatory variables (here, the oceanographic variables), using an extension of multiple linear regression that allows regression of multiple response variables on multiple explanatory variables. Here, only biallelic SNPs were retained, and allele frequencies were Hellinger-transformed prior to RDA [79]. To avoid overdetermination of RDA models with many environmental predictors (Fig B in S1 File), we conducted multiple RDAs on subsets of environmental predictors and reduced the dimensionality of our environmental predictors within sets by first combining them into orthogonal principal components (Tables E–H in S1 File). Specifically, environmental predictors were grouped into four *a priori* sets: (1) all, (2) sea surface, (3) near-bottom and (4) current velocity and temperature predictors (measured at either sea surface or near-bottom). Sets 2 and 3 were chosen to investigate differences in putative adaptation at pelagic and benthic life history stages. Environmental predictor set 4 was chosen for comparison with the existing study on *A*. *californicus* in a different part of the species range, as these variables were strongly correlated with genetic population structure [45]. Predictors were standardized to a mean of 0 and standard deviation of 1 prior to PCA. PCA was performed on each set of predictors, and principal components were retained if their corresponding eigenvalue was above the mean eigenvalue across principal components.

To account for the potential confounding factor of neutral population structure in our RDA, we conducted a complementary partial RDA for each set of predictor variables, in which the effects of spatial variation were partialled out. We used Euclidean distances among collection sites to compute distance-based Morgan’s eigenvector maps (MEMs) using the R package *codep* v.0.9–1 [80], to be used as conditioning variables in partial RDAs [81]. We report the variance inflation factors (VIF) to identify collinearity among spatial variables and environmental predictors, although we note that the effects of such collinearity are addressed in the removal of genetic variation explained by spatial variables in partial RDAs. ANOVA permutation tests for full models and per axis were used to assess the significance of RDA results. For significant models, we identified SNPs putatively involved in local adaption based on the loadings of SNPs in ordination space for significant axes. Specifically, SNPs were classified as putatively adaptive if their loading score was outside of 3 standard deviations of the mean [75].

### Comparing putatively neutral and adaptive differentiation

SNPs were classified as putatively adaptive if they were detected as *F*_*ST*_ outliers using either *BayeScan* or *OutFLANK*, or if they were significantly correlated to environmental predictors using *Bayenv2* or RDA. Once putatively adaptive SNPs were identified, they were used to distinguish a putatively neutral SNP set and a putatively adaptive SNP set.

To address questions related to demographic and selective processes, we used the same methods on putatively neutral and putatively adaptive data sets separately in testing for IBD, estimating dispersal distance, and estimating effective population size per collection site. We estimated effective population size per collection site with *NeEstimator* v.2.1, using the linkage disequilibrium method and default settings [82].

To build hypotheses for the mechanisms underlying adaptive differentiation, potential biological processes associated with putatively adaptive SNPs were identified using *blastx* v.2.5.0 [83] and the UniProt Knowledge Base (Swiss-Prot, manually annotated) [84]. We queried the 2000 *bp* region flanking the SNP, following alignment against the reference genome of *A*. *parvimensis*. Matches with a maximum *e-*value score of 10^−10^ were retained. Gene ontology slim terms for biological processes were retrieved using an adaptation of the Mouse Genome Informatics database, as developed by Gavery and Roberts [85]. Gene ontology slim terms “other biological processes” and “other metabolic processes” were excluded. A particular locus could be associated with multiple gene ontologies, and a particular gene ontology could be associated with multiple gene ontology slim terms for biological processes. All matches were retained.

### Simulations

To contextualize genetic connectivity in terms of migration rate for management, we developed a simulation model using *simuPOP* v.1.1.10.9 [86] in Python v.3.7.3 to determine which population sizes, migration rates, and number of generations of drift reproduced our empirically derived pairwise *F*_*ST*_ results. From an infinitely large ancestral population with global allele frequencies based on our empirical data, we sampled two populations of a specified size and carried those through forward-time simulations with discrete generations, random mating, and no selection. Within each population, two parents were selected at random with replacement to produce one offspring, leading to a random distribution of reproductive success, and allowing for census size to approximate effective population size. The model was parameterized using empirical global allele frequencies for putatively neutral SNPs and simulations were run for each combination of effective population size (500, 2500, 10,000) and migration rate (0.01%, 0.03%, 0.1%, 0.3%, 1%, 3%, 10%, 30%). Additionally, simulations were run for 10 (short-term), 100 (medium-term), and 1,000 (long-term) generations of drift. The long-term option was chosen to approximate equilibrium conditions, although true equilibrium depends on population history and may not be reached in some wild populations within 1,000 generations. Pairwise *F*_*ST*_ was calculated after the pre-determined number of generations of drift had elapsed and averaged across 5 replicates for each parameter combination.

## Results

### Sequencing

After removing 33 (9% of 358 sequenced individuals) poorly sequenced individuals, the average number of reads per individual was 1.76M (standard deviation (SD) = 0.787M). The *dDocent* assembly produced 6,738,423 variant sites, and 2,075 SNPs were ultimately retained after filtering (Table I in S1 File). All individuals included in the *dDocent* assembly were retained after filtering for missing data across SNPs, resulting in an average of 36 individuals per population (SD = 13.7) (Table 1). Genotyping error was estimated to be 1.4% among the 14 replicated individuals. All errors were mismatches of a single allele. Errors were distributed fairly evenly across SNPs, with one error at 246 SNPs, two errors at 19 SNPs, three errors at 7 SNPs, and four errors at one SNP, across replicated individuals. After all filtering steps were completed, the mean missing data per individual and per site was 4.9%, 0.5% of SNPs were multiallelic, and 194 SNPs were out of HWE in one collection site (and 0 SNPs in two or more collection sites) when applying the same HWE filtering method (i.e., FDR approach, *q*-value = 0.05).

### Patterns in genetic diversity

**Fig 2:**
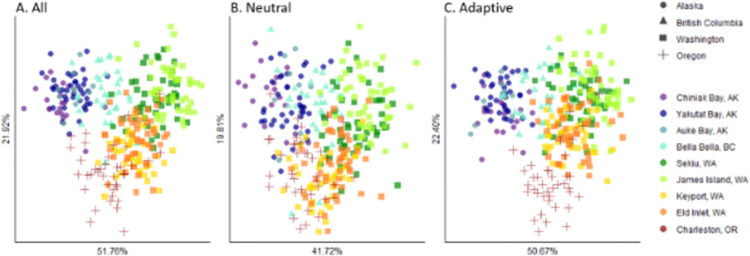
Discriminant analyses of principal components using all SNPs (A), putatively neutral SNPs (B), and putatively adaptive SNPs (C). Each plot represents the first two discriminant functions. For each plot, axes are labeled with the proportion of among-population variance explained by that discriminant function. Points represent individuals and are colored by collection site, matching the colors of collection sites in the map (Fig 1). Point shapes differ by state to highlight regional effects.

Global population differentiation was highly significant using all SNPs (global *F*_*ST*_ = 0.0068, 95% CI [0.005, 0.0105]; global genic differentiation test, *p* < 0.001), although clustering analyses with *ADMIXTURE* provided the strongest support for the model with a single underlying population (Fig C in S1 File). For the DAPC using all SNPs, we retained 53 PCs based on the optimization algorithm and observed clustering by collection site (Fig 2). The DAPC showed evidence for separation of Alaska, British Columbia, Washington, and Oregon sites, with some evidence for separation between sites inside and outside of the Salish Sea and between sites north and south of Admiralty Inlet within the Salish Sea. Permutation test results from AMOVAs demonstrated significant population structure for the NPC grouping using all SNPs (Table 2), suggesting that the North Pacific Current is an oceanographic barrier to dispersal in *A*. *californicus*. Significant population structure for the State grouping was also detected using permutation test results from AMOVAs using all SNPs. The State grouping also had the highest among-group differentiation compared to within-group differentiation (*Φ*_*CT /*_ *Φ*_*SC*_).

**Table 2:** Results of hierarchical population structure, based on AMOVA results.

*Grouping*	*SNPs*	*Φ*_*CT*_	*Φ*_*SC*_	*Φ*_*CT /*_ *Φ*_*SC*_
NPC	All	0.0040*	0.0043*	0.944
	Neutral	0.0016*	0.0020*	0.804
	Adaptive	0.0253*	0.0247*	1.024
Victoria Sill	All	0.0012	0.0056*	0.207
	Neutral	0.0006	0.0025*	0.234
	Adaptive	0.0063	0.0334*	0.187
Admiralty Inlet	All	0.0007	0.0059	0.123
	Neutral	0.0005	0.0026	0.197
	Adaptive	0.0026	0.0357	0.074
All barriers	All	0.0016	0.0052*	0.308
	Neutral	0.0012*	0.0021*	0.585
	Adaptive	0.0052*	0.0335*	0.155
State	All	0.0045*	0.0032*	1.424
	Neutral	0.0015*	0.0018*	0.833
	Adaptive	0.0313*	0.0154*	2.032

Column Grouping contains four groupings considered with AMOVA: North Pacific Current (NPC), Victoria Sill, Admiralty Inlet, State, and All Barriers. Column SNPs refers to which data set was used in the AMOVA: all SNPS (All), putatively neutral SNPs (Neutral), or putatively adaptive SNPs (Adaptive). Variations among groups (Φ_CT_) and among collection sites within groups (Φ_SC_) reported, and significance of permutation tests noted with *: p < 0.05. The ratio of Φ_CT /_ Φ_SC_ also reported.

**Fig 3:**
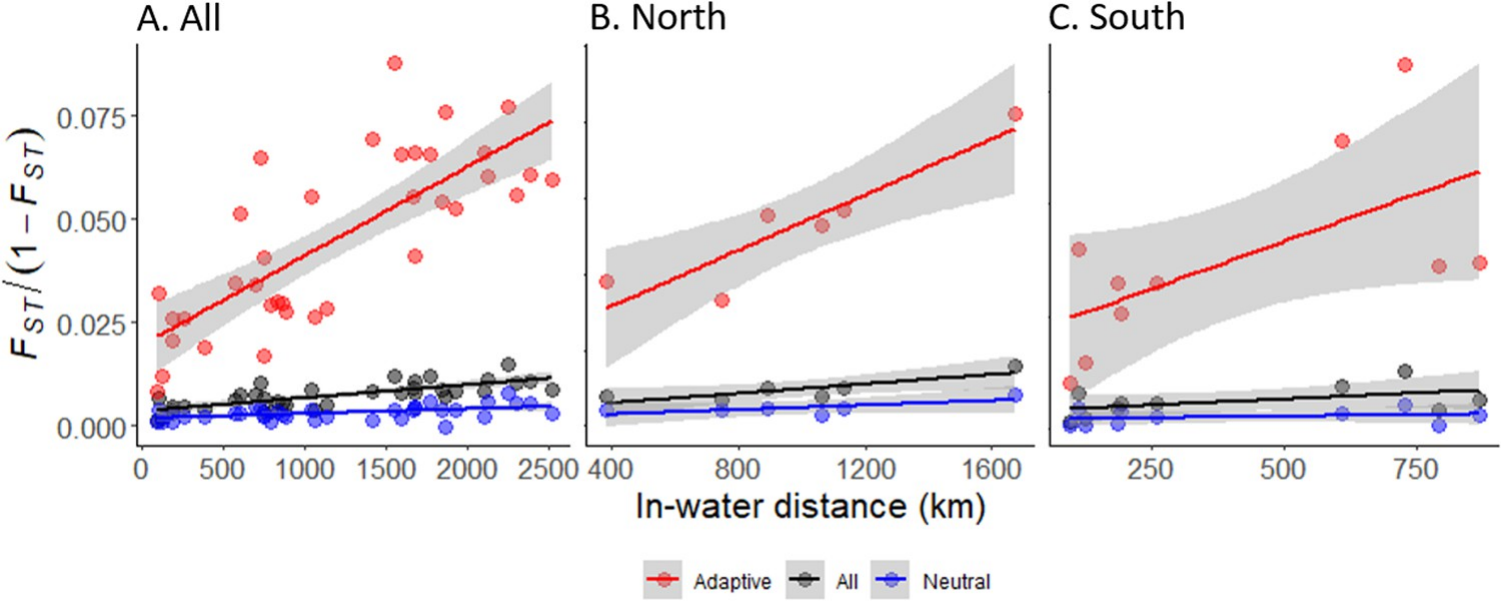
Test for isolation by distance for all collection sites (A), northern collection sites (B), and southern collection sites (C). Correlation between in-water distance and linearized F_ST_ using all SNPs in black, putatively neutral SNPs in blue, and putatively adaptive SNPs in red.

A Mantel test revealed significant positive correlations (adjusted *R*^*2*^ = 0.5622, *p* < 0.001, *y* = (3.2*10^−6^)*x + 3.5*10^−3^) between pairwise linearized *F*_*ST*_ using all SNPs and in-water distance (Fig 3A). This IBD was also evident in the northern part of the range (i.e., Alaska and BC, Fig 3B; adjusted *R*^*2*^ = 0.8433, *p* < 0.05, *y* = (3.1*10^−6^)*x + 1.8*10^−3^) with a remarkably similar slope, but it was not significant in the southern samples (i.e., Washington and Oregon, Fig 3C; adjusted *R*^*2*^ = 0.5009, *p* > 0.05, *y* = (4.3*10^−6^)*x + 3.2*10^−3^). Assuming an effective population density between 100 and 10,000 adults per *km*, this slope suggested a mean dispersal distance per generation of 2 to 20 *km* (Table 3).

**Table 3:** Dispersal distance results.

	Dispersal distance (*km*)		
Density	All	Neutral	Adaptive
1	198	337	75
10	63	107	24
100	20	34	8
1,000	6	11	2
10,000	2	3	1
100,000	1	1	0

Mean dispersal distances per generation in km, estimated using all, putatively neutral, and putatively adaptive SNPs, depending on effective population density (adults / km).

Of all 36 pairwise site comparisons using all SNPs (Table 4), 24 revealed significant genic differentiation (*p* < 0.05), with 8 of the 12 not statistically significant tests corresponding to collection site pairings with the small collection from Auke Bay, AK (Table 1). Only one pairwise site comparison (Keyport and Eld Inlet, WA) yielded a pairwise *F*_*ST*_ value that contained 0 in the 95% confidence interval (Table J in S1 File). The largest pairwise *F*_*ST*_ values were found among collection sites from the northern (Alaska and British Columbia) and southern (Washington and Oregon) parts of the species range (*F*_*ST*_ = 0.005–0.015). Notably, pairwise differentiation between collection sites within Washington was broader in range and at times greater than that within Alaska, despite substantially smaller in-water distances (mean = 162 *km*, SD = 62 *km* in WA and mean = 731 *km*, SD = 339 in AK).

**Table 4:** Pairwise F_ST_ and genic differentiation test results.

	CB_AK	YB_AK	AB_AK	BB_BC	SK_WA	JI_WA	KP_WA	EI_WA	CH_OR
CB_AK	-				*	*	*	*	*
YB_AK	0.004	-		*	*	*	*	*	*
AB_AK	0.004	0.004	-						
BB_BC	0.008	0.005	0.005	-	*	*	*	*	*
SK_WA	0.011	0.009	0.008	0.006	-		*	*	*
JI_WA	0.015	0.012	0.012	0.007	0.002	-	*	*	*
KP_WA	0.010	0.009	0.008	0.007	0.004	0.006	-		*
EI_WA	0.011	0.008	0.011	0.006	0.005	0.005	0.001	-	*
CH_OR	0.009	0.008	0.007	0.009	0.008	0.010	0.004	0.005	-

Pairwise F_ST_ in the lower left and pairwise genic differentiation test results in the upper right of the table (*: p < 0.05). Site abbreviations provided in Table 1.

Mean expected heterozygosity and proportion of polymorphic SNPs did not vary substantially across collection sites, apart from a reduced proportion of polymorphic SNPs in Auke Bay and Chiniak Bay, AK (Table 1). Observed heterozygosity decreased and *F*_*IS*_ increased from North to South, while expected heterozygosity was similar across the range (Table 1). All collection site *N*_*e*_ estimates were large or infinite, with infinite upper confidence limits, using both all putatively neutral SNPs and only those with a minor allele frequency of at least 5% (Table 5).

**Table 5:** Linkage desquilibrium N_e_ estimates with 95% confidence intervals.

	*Pcrit*_*0*.*05*_		*Pcrit*_*0*.*0*_	
*Site*	*Estimate*	*95% CI*	*Estimate*	*95% CI*
Chiniak Bay, AK	1170.4	319.6—Infinity	40375.1	389.6—Infinity
Yakutat Bay, AK	6368.8	1088.9—Infinity	21798.0	1223.1—Infinity
Auke Bay, AK	Infinity	312.6—Infinity	Infinity	312.6—Infinity
Bella Bella, BC	1063.5	238.1—Infinity	1453.5	248.2—Infinity
Sekiu, WA	16057.9	2365.8—Infinity	21545.6	2550.8—Infinity
James Island, WA	181563.8	2407.7—Infinity	25852.1	2949.4—Infinity
Keyport, WA	Infinity	4596.7—Infinity	Infinity	5719.8—Infinity
Eld Inlet, WA	5117.3	1605.0—Infinity	5683.6	2055.4—Infinity
Charleston, OR	Infinity	2645.8—Infinity	Infinity	3272.6—Infinity

Confidence intervals estimated by jackknifing across samples. Pcrit is the minimal allele frequency to retain a locus in the analysis. Both Pcrit_0.05_ (0.05%) and Pcrit_0.0_ (0%) were included because rare alleles can affect N_e_ estimates.

### Adaptive differentiation

**Fig 4:**
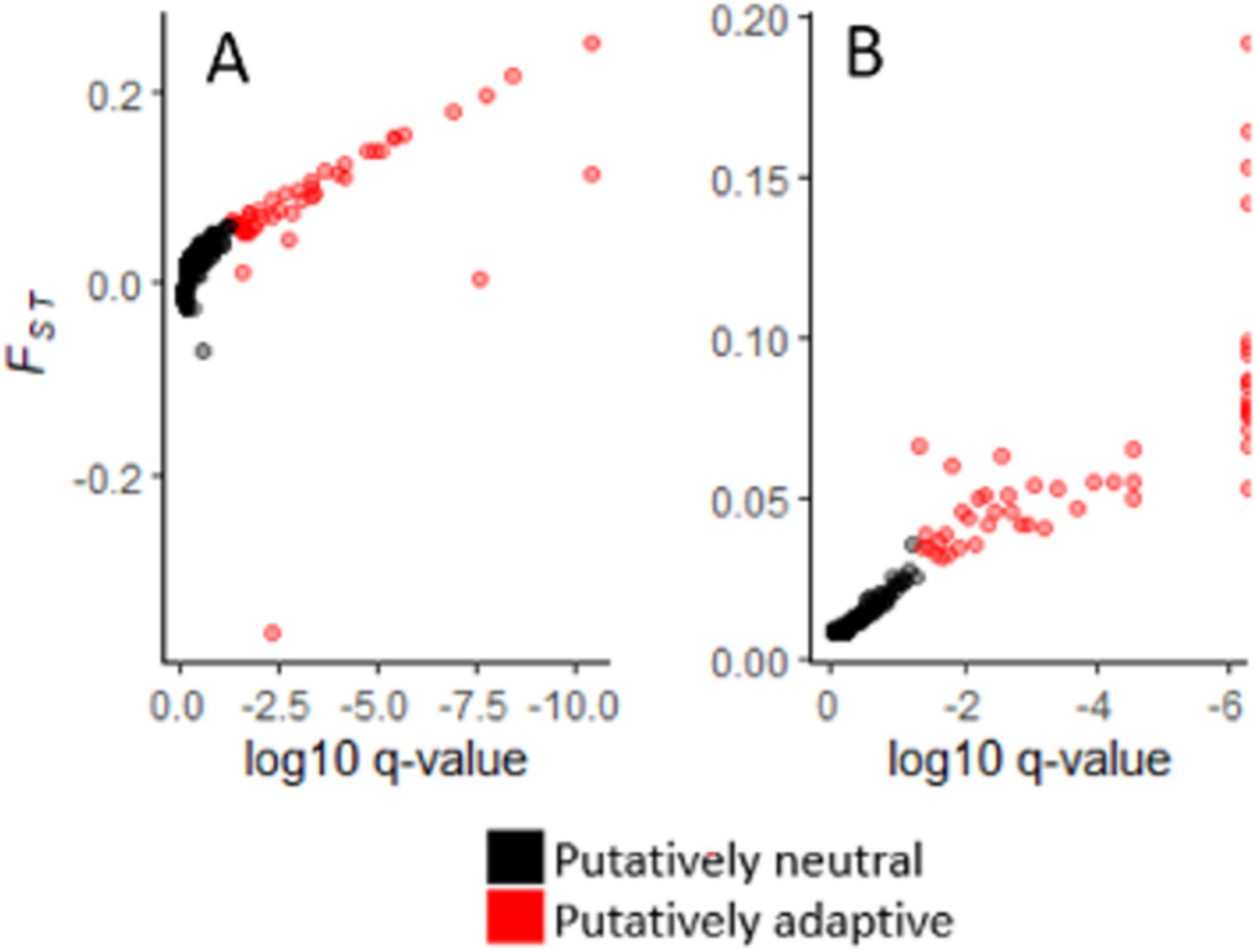
Results of F_ST_ outlier detection methods. Log_10_ q-value by F_ST_ for outlier detection methods, using results generated by OutFLANK (n = 46) in panel A and BayeScan (n = 50) in panel B. Each point represents a single SNP, colored by whether it was detected as an outlier (red) or not (black).

The *F*_*ST*_ outlier SNP detection methods identified 60 (2.9% of 2,075) SNPs as putatively adaptive (Fig 4), with 50 identified by *BayeScan*, 46 identified by *OutFLANK*, and 36 SNPs overlapping across both methods.

Using *Bayenv2*, 181 SNPs (8.7% of 2,075) were identified as putatively adaptive. Each of these SNPs was correlated with an average of 2.5 environmental predictors and at least one SNP was correlated with each of the 29 environmental predictors (Table K in S1 File). The five environmental variables with the most associated SNPs included mean salinity, mean nitrate, temperature range, and pH, all at the sea surface, and mean temperature at near-bottom (Table 6). Over 60% more SNP-predictor correlations represented environmental variables measured at the surface than at near-bottom, for variables with both measurements: 397 and 615 SNP-predictor correlations with near-bottom and sea surface variables, respectively.

**Table 6:** Univariate gene-environment association results.

*Predictor*	*Depth*	*S*_*C*_	*S*_*P*_	*Biological processes*
Mean salinity	S	38	4	signal transduction (0.259), cell organization and biogenesis (0.207), protein metabolism (0.207), cell cycle and proliferation (0.138), RNA metabolism (0.086), transport (0.069), DNA metabolism (0.034)
Mean nitrate	S	34	4	signal transduction (0.294), cell organization and biogenesis (0.235), developmental processes (0.216), cell cycle and proliferation (0.078), transport (0.078), protein metabolism (0.059), DNA metabolism (0.039)
Temperature range	S	31	2	cell organization and biogenesis (0.387), signal transduction (0.29), RNA metabolism (0.097), developmental processes (0.065), DNA metabolism (0.065), protein metabolism (0.065), stress response (0.032)
pH	S	30	3	cell organization and biogenesis (0.353), developmental processes (0.324), signal transduction (0.265), DNA metabolism (0.059)
Mean temperature	B	27	1	signal transduction (0.5), transport (0.5)

Putatively adaptive loci associated with environmental variables from Bayenv2 for five environmental predictors (column Predictor) with most correlated SNPs. Column Depth refers to whether the environmental variable is measured at sea surface (S) or mean bottom depth (B). Column S_C_ contains the number of SNPs with evidence of correlation to the row’s variable. Column S_P_ contains the number of correlated SNPs that also had matching gene ontology SLIM terms for biological processes. Column Biological processes contains the associated biological processes from matching GO slim terms in order of decreasing frequency, with the proportion of matches per process noted in parentheses.

**Fig 5:**
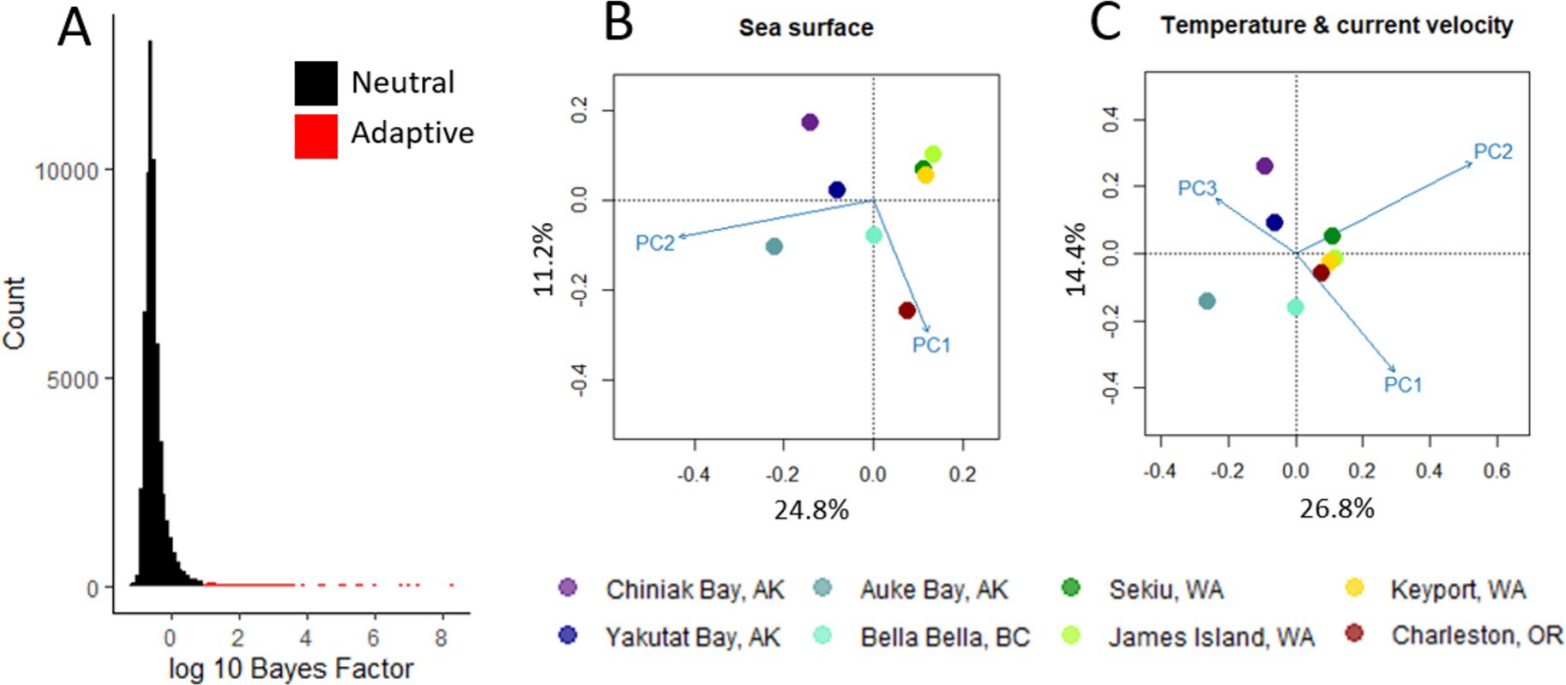
Summary of the results of univariate (Bayenv2) and multivariate (RDA) gene-environment associations. Panel A is a histogram of log10 Bayes Factors for each combination of SNP and environmental predictor. Panel B and C are RDA biplots of the first two RDA axes, with predictors as vectors and sites as points in ordination space, using Type 1 scaling, a scaling method appropriate for questions related to distance among objects. Panel B is of the retained model using sea surface predictors, and panel C is of the retained model using temperature and current velocity predictors. For each RDA biplot, the proportion of variance explained by each RDA axis is labeled on the axis.

After removing 10 multiallelic SNPs, we retained 2,065 biallelic SNPs for use in redundancy analyses (RDA). We retained two RDA models (out of 8) for statistical significance: the model using sea surface predictors was marginally significant at the full model level (ANOVA, *p* = 0.063) and for the first axis (ANOVA, *p* = 0.052), and the model using temperature and current velocity predictors (at sea surface or near-bottom) was significant at the full model level (ANOVA, *p* = 0.034) and for the first axis (ANOVA, *p* = 0.035). For the RDA using sea surface predictors, the first RDA axis explained 24.8% of the variance, and PC 2 had the greatest loading on the first RDA axis (Table L in S1 File). The three predictors with the greatest loadings on PC 2 included pH, mean salinity, and temperature range (Table E in S1 File). For the RDA using temperature and current velocity predictors, the first RDA axis explained 26.8% of the variance, and PC 2 had the greatest loading on the first RDA axis (Table M in S1 File). The three predictors with the greatest loading on PC 2 included temperature range at the sea surface and near-bottom, and minimum temperature at the near-bottom (Table H in S1 File). Collection site clustering patterns were similar among RDA biplots (Fig 5), including the Salish Sea collection sites clustering tightly together and North-South separation driven by the first RDA axis in each model. Charleston, Oregon clustered more closely with the Salish Sea collection sites in the model using temperature and current velocity predictors (Fig 5). We identified 32 putatively adaptive SNPs based on loadings of significant (and marginally significant) RDA axes, with 24 SNPs identified from the RDA using sea surface predictors and 29 from the RDA using temperature and current velocity predictors. No partial RDAs were significant. Spatial variables used in partial RDAs covaried with predictor variables in most models, measured with variance inflation factors (Table 7). The environmental predictor loadings for retained PCs in all models and a correlation matrix of environmental predictors can be found in Tables D–H in S1 File.

**Table 7:** Results of the redundancy analyses (RDA).

*RDA*	*Predictors*	*Partial*	*R* ^*2*^_*adj*_	*p*_*full*_	*p*_*axis*_	*VIF > 10*
1	all	Yes	0.094	>0.1	>0.1	1 MEM & 2 PCs
2	all	No	0.07	>0.1	>0.1	-
3	surface	Yes	0.16	>0.1	>0.1	2 MEMs & 2 PCs
4	surface	No	0.11	0.063	0.052–0.606	-
5	bottom	Yes	-0.081	>0.1	>0.1	-
6	bottom	No	-0.0007	>0.1	>0.1	-
7	cv & temp	Yes	0.087	>0.1	>0.1	1 MEM & 2 PCs
8	cv & temp	No	0.14	0.034	0.035–0.695	-

Column RDA assigns a number to each RDA for reference; Predictors refers to the set of environmental predictors used in the RDA, where all refers to all 29 predictors, surface refers to sea surface, bottom refers to mean bottom depth, and cv & temp refers to current velocity and temperature predictors; Partial refers to whether spatial variables were conditioned in a partial RDA; R^2^_adj_ refers to the adjusted R^2^ value; p_full_ and p_axis_ refer to the p-value from the ANOVA on the full model and for axes, respectively; and VIF > 10 refers to whether any variance inflation factors (VIF) were over ten, suggesting substantial co-variance among explanatory variables, where MEM refers to spatial variables (Morgan’s eigenvector maps) and PC refers to principal components.

In total, we identified 211 (10.2% of 2,075 total SNPs) putatively adaptive SNPs, of which 41 (19.4% of 211 putatively adaptive SNPs) were detected with either *F*_*ST*_ outlier approaches and at least one gene-environment association approach and 134 (63.5% of 211) were only identified by *Bayenv2* (Fig D in S1 File).

Only 19 (9% of 211) putatively adaptive SNPs matched to gene ontologies, and 15 (79% of 19) matched to more than one gene ontology. Of the SNPs identified as putatively adaptive using *Bayenv2*, 15 SNPs matched to gene ontologies (Table 6, Table K in S1 File). Of these 15 SNPs, three were also identified using *Bayescan* and *OutFLANK* and one was also identified using *Bayescan*, *OutFLANK*, and RDA. An additional four SNPs were also identified as putatively adaptive using only *F*_*ST*_ outlier methods, with the most represented biological processes including DNA metabolism, cell organization and biogenesis, and developmental processes. For comparison, the top five most frequent biological processes in the database in descending order are 1) developmental processes, 2) cell organization and biogenesis, 3) transport, 4) protein metabolism, and 5) stress response (Table N in S1 File).

### Neutral vs. putatively adaptive differentiation

DAPC and AMOVA revealed higher differentiation but similar spatial patterns for putatively adaptive SNPs compared to putatively neutral SNPs (Table 2, Fig 2). We retained 53 and 26 PCs for the DAPC using putatively neutral SNPs and putatively adaptive SNPs, respectively, based on the optimization algorithm. Clustering patterns using putatively neutral and putatively adaptive SNPs reflected those using all SNPs, with more distinct clustering using putatively adaptive SNPs (Fig 2). Permutation test results from AMOVAs demonstrate significant population structure for NPC and State groupings, using putatively neutral and adaptive SNPs (Table 2). The ratio of among-group variation to within-group variation (*Φ*_*CT /*_ *Φ*_*SC*_) was higher using putatively adaptive SNPs for the State and NPC groupings, and higher using putatively neutral SNPs for the Victoria Sill and Admiralty Inlet groupings. Mantel tests for correlation among linearized *F*_*ST*_ and in-water distance were significant using putatively neutral SNPs (adjusted *R*^*2*^ = 0.2217, *p* < 0.01, *y* = (1.1*10^−6^)*x + 1.7*10^−3^) and putatively adaptive SNPs (adjusted *R*^*2*^ = 0.5786, *p* < 0.001, *y* = 2.2*10^−5^ + 2.0*10^−2^) (Fig 3A). Similar to analyses with all SNPs, slopes for adaptive SNPs were similar across the entire range and in Alaska and BC (adjusted *R*^*2*^ = 0.9187, *p* < 0.05, *y* = (1.7*10^−5^)*x + 8.8*10^−3^), and not significant in samples from Oregon and Washington (adjusted *R*^*2*^ = 0.621, *p* > 0.05, y = (3.1*10^−5^)*x* + 1.6*10^−2^; Fig 3). Mantel tests using putatively neutral SNPs were not significant when performed only on northern collection sites (adjusted *R*^*2*^ = 0.6757, *p* > 0.05, *y* = (1.5*10^−6^)*x + 9.5*10^−4^) or southern collection sites (adjusted *R*^*2*^ = 0.2631, *p* > 0.05, *y* = (1.1*10^−6^)*x + 1.8*10^−3^). Correlation among linearized *F*_*ST*_ and in-water distance was strongest and the slope of the linear regression was greatest using putatively adaptive SNPs: the slope of the linear regression using putatively adaptive SNPs was nearly 7 and 20 times greater than the slopes using all SNPs and putatively neutral SNPs, respectively. Assuming a population density between 100 and 10,000 adults per *km*, we estimated mean dispersal distance to be between 3 and 34 *km* using putatively neutral SNPs and between 1 and 8 *km* using putatively adaptive SNPs (Table 3).

### Simulations

**Fig 6:**
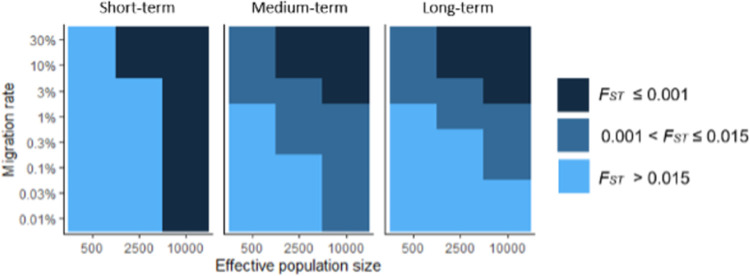
Results of simulations that investigated demographic conditions that reproduced empirical F_ST_ results. Tile maps represent pairwise F_ST_ for each combination of effective population size, migration rate, and number of generations of drift elapsed. Short-term (left) represents results from 10 generations of drift, Medium-term (middle) from 100 generations of drift, and Long-term (right) from 1,000 generations of drift. Tile color is divided into three groups based on the scale of F_ST_: smaller than the observed F_ST_ values in this study, within the range of observed pairwise F_ST_, and greater than the observed pairwise F_ST_.

Simulations revealed that in an idealized two-population system, simulated pairwise *F*_*ST*_ within the range observed in this study occurred in the long-term (1,000 generations since divergence) with small effective population size (*N*_*e*_ = 500) and high migration (*m* ≥ 3%), with moderate effective population size (*N*_*e*_ = 2,500) and moderate migration (1% ≥ *m* ≥ 3%), and with large effective population size (*N*_*e*_ = 10,000) and small to moderate migration (0.1% ≥ *m* ≥ 1%). Most simulations that produced pairwise *F*_*ST*_ estimates within the range observed in this study were cases with moderate to large effective population size (*N*_*e*_ ≥ 2,500) and migration rates below 10%. Simulated pairwise *F*_*ST*_ remained below the lowest empirically observed pairwise *F*_*ST*_ in this study in the long-term (1,000 generations) only when as effective population size was at least moderate (*N*_*e*_ ≥ 2,500) and migration rate was high (*m* ≥ 10%). Under realistic conditions (e.g., *N*_*e*_ = 10,000 and 1,000 generations since divergence; our *N*_*e*_ estimates were large and unbounded), simulated pairwise *F*_*ST*_ was within the range of empirical pairwise *F*_*ST*_ when migration rates were 0.1–1.0% (Fig 6).

## Discussion

In this study, we quantified patterns of population differentiation in *A*. *californicus* and investigated potential drivers of differentiation from Alaska to Oregon, representing more than half of the species’ range and regions not yet surveyed. We observed population genetic structure at both fine- and broad-scales, likely driven by limited dispersal and local adaptation. Notably, we found detectable differentiation at small scales within the Salish Sea, co-varying signals of adaptive and neutral differentiation, and a latitudinal pattern in genetic diversity. Estimates of effective population size were large. Using simulations, we found that migration rates may be below 1% if populations of *A*. *californicus* are large (*N*_*e*_ = 10,000) and have undergone many (1,000) generations of drift after splitting from a single ancestral population. These estimates corresponded well to average dispersal distances per generation from isolation-by-distance patterns, which were on the scale of dozens of kilometers despite the long pelagic larval duration.

### Broad- and fine-scale population genetic structure and potential drivers

Broad-scale population structure that we observed can be described by two patterns: differentiation across the bifurcation zone of the North Pacific Current and isolation-by-distance. Many species of marine invertebrate exhibit population differentiation along a latitudinal gradient, with the region between Alaska and Oregon as a known region of divergence [87, 88]. Xuereb et al. [45] posit that the genetic break observed in *A*. *californicus* in British Colombia is due to limited dispersal across the bifurcation zone of the North Pacific Current. The North Pacific Current has also been identified as an oceanographic barrier to gene flow in the Bat Star, *Patiria miniata* [44, 89], and the Rosethorn Rockfish, *Sebastes helvomaculatus* [90]. In addition to this break, Xuereb et al. [22] detected IBD in *A*. *californicus* along the coast of British Columbia [22], but only across the entire study range, not within the southern and northern population component, suggesting that IBD was an artefact of population subdivision rather than a signal of limited dispersal in a continuous population [91]. Here, IBD was found across the entire range from Alaska to Oregon as well as in the section north of the North Pacific current bifurcation, but not among more southern samples. IBD was also found in other species of sea cucumbers including *Holothuria edulis* [92], *H*. *scabra* [93], and *H*. *nobilis* [94]. Our results did not provide evidence for limited dispersal across the Victoria Sill or the sills at Admiralty Inlet, despite their potential roles in shaping population structure in other marine species [40, 41, 95, 96]. Although biologically not meaningful, the State grouping yielded the greatest among-group variation, likely because it captured the effects of isolation-by-distance and the divergence across the NPC bifurcation zone. While this result may also be caused by our specific sampling design (e.g., only one sample from BC and Oregon, all Washington samples within the Salish Sea), it lends support to current management by individual state agencies.

Additionally, we found a subtle pattern of decreasing observed heterozygosity, constant expected heterozygosity and increasing *F*_*IS*_ from north to south, a pattern that can also be observed in the data of Xuereb et al. [65]. In temperate species, genetic diversity usually declines polewards as a consequence of successive founder events during postglacial recolonization [97], opposite to the pattern observed here. Higher *F*_*IS*_ values in the south could be an effect of selection against heterozygotes caused by high genetic load of deleterious mutations, as commonly observed in bivalves [98], but also other highly fecund marine species such as echinoderms [99]. Such selection may be more severe in warmer waters for cold adapted species, as environmental stress increases selection against deleterious mutations [100], thus explain higher *F*_*IS*_ values in southern samples. Heterozygote deficiencies can also be caused by mixtures (Wahlund effect) of populations, cohorts or local demes [53]–although population mixture appears unlikely given generally weak population structure, mixtures of cohorts in mixed-age samples produced by sweepstake recruitment could produce heterozygote deficiencies [101] in the south. Population mixtures could also be caused by temporally varying recruitment sources or selection [102, 103]. Alternative explanations such as inbreeding or assortative mating appear less likely because estimates of effective population size all had infinite upper confidence limits, Alaska supports larger sea cucumber fisheries compared to southern regions [104], and expected heterozygosity was similar between northern and southern samples.

Notably, we found that geographic distance was more strongly correlated to genetic distance among collection sites using putatively adaptive SNPs than using putatively neutral SNPs, though neither relationship was significant in the southern samples. The higher slope of the relationship resulted in lower estimates of mean dispersal estimates using putatively adaptive SNPs than putatively neutral SNPs. This finding suggests that selection gradients may reduce effective dispersal distances, leading to stronger signals in isolation-by-distance from neutral processes alone [105]. One exception may be the Charleston, Oregon collection site, which clusters more distinctly from other collection sites using putatively adaptive SNPs and is positioned more closely to the Salish Sea collection sites in the RDA biplot for temperature and current velocity predictors than the biplot for sea surface predictors. The difference in RDA biplots suggests different patterns in adaptive differentiation, dependent on environmental predictors. Lastly, co-variation between neutral and adaptive differentiation could also represent a false positive result for gene-environment association. Isolation-by-distance, as we found in this study, can increase the chance of a false negative result for gene-environment association if the environmental predictor is spatially auto-correlated [106].

At finer scales, we found detectable differences among most collection site pairs and between collection sites close together as 110 *km* (in-water distance) apart, consistent with earlier findings on differentiation as close as 60 *km* in *A*. *californicus* [22] and 100 *km* apart in the Tar-spot Sea Cucumber, *Cucumaria pseudocurata* [107]. The sample from Auke Bay, Alaska was an outlier across multiple analyses, likely due to stochasticity associated with smaller sample size, and leading to most insignificant pairwise genic differentiation tests and a lower proportion of polymorphic SNPs. Pairwise differentiation was lower in Alaska despite larger in-water distances between collections, possibly because of longer larval periods in colder Alaskan waters resulting in more extensive dispersal. Colder temperatures led to slower larval growth in laboratory experiments in the sister species, the Japanese sea cucumber, *A*. *japonicus* [108], and meta-analyses in marine fishes showed longer larval duration, larger dispersal distances, and higher *F*_*ST*_ in high latitude species [109]. Alternatively, greater genetic differentiation among populations within Washington compared to Alaska may point to underlying oceanographic barriers that can shape dispersal in complex and asymmetrical ways. In future work, a denser sampling scheme paired with an oceanographic model could be used to illuminate the potential for seascape features to influence dispersal, particularly within the Salish Sea.

We found evidence for putatively adaptive differentiation, corroborated by multiple lines of investigation. The majority of SNPs identified as putatively adaptive using *F*_*ST*_ outlier detection were identified using both methods and were also identified as putatively adaptive using at least one gene-environment association method. Using *Bayenv2*, we detected many SNPs that were not identified as putatively adaptive compared to redundancy analysis, consistent with expectations that *Bayenv2* may produce more false positives compared to redundancy analysis [75]. The five environmental predictors with the most significantly correlated SNPs were salinity, nitrate, temperature range, and pH at sea surface and mean temperature at near-bottom. Of these, salinity at the sea surface and temperature at the sea bottom were also identified as potential drivers of local adaptation in the same species in British Columbia [45]. Temperature and salinity are significant factors shaping growth and survival in many marine organisms [110] and have been shown to affect growth and survival in *A*. *japonicus* juveniles [111] and growth, survival, locomotory speed, and metamorphosis in *A*. *japonicus* larvae [108]. The greater number of SNPs significantly correlated to sea surface predictor variables using *Bayenv2* and the occurrence of temperature variables as top predictor variables is consistent with RDA results: the significant RDA models were those with environmental predictors at the sea surface and environmental predictors related to temperature and current velocity. Thus, we hypothesize that selection at early life history stages, when larvae are at the sea surface, drives adaptive differentiation in *A*. *californicus*, with salinity, nitrate, pH, and temperature as possible selection factors.

We note that we cannot exclude balanced polymorphism as an alternative explanation to observed putative adaptive differentiation: it is possible that selective mortality at early life stages leads to observed patterns of putative adaptive differentiation in adults (as were sampled here), but that genetic variation is maintained across time through high gene flow [3]. As mentioned above, heterozygote deficiency could be caused by mixtures of cohorts originating from temporally or spatially varying recruitment events undergoing different selection. However, increasing evidence suggests that selection for locally adapted alleles can occur despite high gene flow [7, 112], particularly in species with large effective population sizes.

In generating hypotheses about which environmental predictors are potential drivers of selection, it is prudent to be cautious and highlight 1) spatial auto-correlation of predictors, particularly in light of correlation between geographic distance and genetic distance using putatively adaptive SNPs as observed here, 2) correlation among environmental predictors and 3) the incomplete genome coverage of reduced representation approaches such as RAD sequencing. Almost all environmental predictor sets contained spatially auto-correlated variables, evidenced by high variance inflation factors for spatial variables and environmental predictor variables in partial RDAs (Table 7). Additionally, the correlation among environmental variables adds uncertainty about which predictors are driving patterns in adaptive differentiation. These correlations highlight the limits of these analyses. Finally, although the usefulness of RAD sequencing for identifying adaptive divergence has been questioned [113], RAD sequencing has been used successfully to reveal genomic signatures of selection in many non-model species [114]. Our results are fairly consistent with those of Xuereb et al. [45] and Xuereb et al. [115] even though they sampled genetic and environmental data from different collection sites. Confidence in gene-environment association results may increase if results are further corroborated in future studies, particularly if using alternative methods such as whole genome sequencing. In any case, the use of adaptive genetic variation for practical management applications is currently an issue of debate and needs further research [115].

For the environmental variables with the most correlated SNPs, and for such SNPs with associated biological processes, signal transduction appeared among the most common biological processes. Future research investigating the connections between genotype, phenotype, and observed patterns in adaptive differentiation may start with the potential connection between signal transduction pathways and salinity, nitrate, temperature, and pH. Others have identified signal transduction genes as part of an adaptive response to salinity adaptation and stress in sea cucumbers [116]. In *A*. *japonicus*, high salinity conditions were correlated with downregulation of acetylcholinesterase, the enzyme that terminates signal transduction. Inhibition of this enzyme can lead to excessive stimulation of nerve and muscle tissue, ultimately leading to paralysis and even death in some species [117]. Transcriptomic approaches can be used in future studies of wild populations of *A*. *californicus* across environmental gradients or in common garden experiments with varying environmental treatments to build cases for which environmental factors shape adaptive differentiation and through which physiological pathways.

### Spatial considerations for sustainable management of *A*. *californicus*

Simulation results suggest limited dispersal among collection sites: if effective population sizes were at least 10,000 and separated at least 1,000 generations ago, genetic differentiation suggested migration rates of 0.1–1% (Fig 6). Here, our estimates of effective population size were large with infinite upper confidence limits, demonstrating that populations are likely not small [118]. This level of migration (0.1–1%) may be large enough to prevent genetic isolation, but small enough to lead to demographic independence [119]. This has implications for spatial management of wild *A*. *californicus*, though those implications may differ depending on the management aim. For example, marine protected areas for *A*. *californicus* may need to operate at smaller scales than expected from signals of genetic differentiation at broad scales, to maintain demographic connectivity and prevent overexploitation of local populations. Designation of management units for fisheries may need to occur at similar scales, because fishery management units are used to prevent overexploitation of local populations [12], largely a demographic concern. On the other hand, management units designed to protect wild populations as a genetic resource for aquaculture aim to prevent the loss of genetic diversity and fitness of wild populations due to broodstock collection and farm escapees, and may thus be better defined at a larger geographic scale than demographic units for fisheries management.

Gradual differentiation associated with the IBD pattern of population structure poses a unique challenge in determining the spatial scale of management units for fisheries and aquaculture. Without distinct boundaries between populations, it is difficult to delineate management units. However, in species with IBD, genetic variation can be preserved even if arbitrary boundaries are chosen [120]. In *A*. *californicus*, it is likely that IBD and oceanographic barriers such as the NPC shape population structure, such that variation may be gradual in many parts of the range at fine and broad scales, but that oceanographic barriers may create distinct boundaries at fine scales [22]. Assessing population structure at finer scales in areas of interest may illuminate oceanographic barriers to dispersal not yet detected and facilitate delineation of management units. In the absence of further sampling, it may be assumed from a precautionary standpoint that distances over which detectable variation can occur (~100 *km*) may be a useful starting point for defining boundaries in delineation of management units, particularly for fishery management units. As populations are further subdivided for management based on genetic data, decision-makers must also consider the risk of reduced precision associated with the need to complete stock assessments for more stocks with less data [121]. From a less precautionary standpoint, state and provincial boundaries may also be used in the absence of further data, as clustering patterns and partitioning of variance using AMOVAs suggested that this hierarchical grouping explained the most genetic variation of considered groupings. Due to observed isolation-by-distance, it is likely that additional samples will uncover differentiation at smaller scales than states and provinces.

## Supporting information

S1 File (PDF)
