# Supplementary material for: Population structure and adaptive differentiation in the sea cucumber Apostichopus californicus and implications for spatial resource management
Source: PLoS One. 2023 Mar 16;18(3):e0280500. doi: 10.1371/journal.pone.0280500 (PMC10019739; doi:10.1371/journal.pone.0280500)
Supplement: S1 File — (PDF) [file pone.0280500.s001.pdf]

Supplementary Information

Table A. Comparing de novo *and with reference assembles using Pairwise* F_{ST} *and genic differentiation tests.*

De novo	CB_AK	YB_AK	AB_AK	BB_BC	SK_WA	JI_WA	KP_WA	EI_WA	CH_OR
CB_AK	-			*	*	*	*	*	*
YB_AK	0.0036	-		*	*	*	*	*	*
AB_AK	0.0043	0.0037	-						
BB_BC	0.0076	0.006	0.0038	-	*	*	*	*	*
SK_WA	0.0122	0.0094	0.0075	0.0049	-		*	*	*
JI_WA	0.0139	0.0114	0.0099	0.0061	0.0014	-	*	*	*
KP_WA	0.0101	0.0088	0.0074	0.006	0.0033	0.0056	-		*
EI_WA	0.0108	0.0098	0.0088	0.0062	0.004	0.0048	0.0006	-	*
CH_OR	0.011	0.011	0.0089	0.0093	0.0081	0.0109	0.0036	0.0045	-

Reference	CB_AK	YB_AK	AB_AK	BB_BC	SK_WA	JI_WA	KP_WA	EI_WA	CH_OR
CB_AK	-				*	*	*	*	*
YB_AK	0.004	-		*	*	*	*	*	*
AB_AK	0.004	0.004	-						
BB_BC	0.008	0.005	0.005	-	*	*	*	*	*
SK_WA	0.011	0.009	0.008	0.006	-		*	*	*
JI_WA	0.015	0.012	0.012	0.007	0.002	-	*	*	*

KP_WA	0.010	0.009	0.008	0.007	0.004	0.006	-		*
EI_WA	0.011	0.008	0.011	0.006	0.005	0.005	0.001	-	*
CH_OR	0.009	0.008	0.007	0.009	0.008	0.010	0.004	0.005	-

Pairwise F_{ST} *(lower left) and genic differentiation test results (upper right, *:* $p < 0.05$*) for* A. californicus*, comparing* de novo *assembly (top) with reference-guided assembly (bottom) to demonstrate similarity in patterns. Global* F_{ST} *in both* de novo *and reference-guided assembly = 0.0068. Collection sites are represented by site code (Table 1) and the color of the circle next to each collection snite name matches the colors for each collection site in the map (Fig 1). Cells are shaded from white to dark green, representing low to high pairwise* F_{ST}.

***Table B. Comparing* de novo *and with reference assembles using with expected heterozygosity, observed heterozygosity, and proportion of polymorphic SNPs by collection site*.**

	De novo			*With reference*		
Collection site	H_E	H_O	S_P	H_E	H_O	S_P
Chiniak Bay, AK	0.273	0.256	0.946	0.278	0.251	0.932
Yakutat Bay, AK	0.274	0.253	0.985	0.279	0.250	0.973
Auke Bay, AK	0.271	0.242	0.865	0.275	0.240	0.837
Bella Bella, BC	0.278	0.235	0.969	0.286	0.232	0.951
Sekiu, WA	0.273	0.241	0.989	0.279	0.238	0.977
James Island, WA	0.271	0.235	0.987	0.277	0.233	0.968
Keyport, WA	0.271	0.237	0.987	0.278	0.233	0.964
Eld Inlet, WA	0.273	0.236	0.987	0.280	0.234	0.970
Charleston, OR	0.271	0.237	0.982	0.277	0.233	0.968

H_E *refers to mean expected heterozygosity,* H_O *refers to mean observed heterozygosity, and* S_P *refers to the proportion of SNPs that are polymorphic.*

Table C. Environmental predictor codes and descriptions from Bio-Oracle (BO) and Bio-Oracle2 (BO2) databases.

Code	Description
BO_calcite	Calcite concentration indicates the mean concentration of calcite (CaCO3) in oceans.
BO_ph	Measure of acidity in the ocean.
BO_bathymean	Average depth of the seafloor
BO2_curvelmax_bdmean	Maximum sea water velocity at mean bottom depth
BO2_curvelmean_bdmean	Mean sea water velocity at mean bottom depth
BO2_curvelmin_bdmean	Minimum sea water velocity at mean bottom depth
BO2_curvelrange_bdmean	Range of the sea water velocity at mean bottom depth
BO2_dissoxmean_bdmean	Mean mole concentration of dissolved molecular oxygen in sea water at mean bottom depth
BO2_phosphatemean_bdmean	Mean mole concentration of phosphate in sea water at mean bottom depth
BO2_nitratemean_bdmean	Mean mole concentration of nitrate in sea water at mean bottom depth
BO2_tempmax_bdmean	Maximum sea water temperature at the bottom at mean bottom depth
BO2_tempmean_bdmean	Mean sea water temperature at the bottom at mean bottom depth
BO2_tempmin_bdmean	Minimum sea water temperature at the bottom at mean bottom depth
BO2_temprange_bdmean	Range of the sea water temperature at the bottom at mean bottom depth
BO2_ppmean_bdmean	Mean net primary productivity of carbon at mean bottom depth
BO2_salinitymean_bdmean	Mean sea water salinity at the bottom at mean bottom depth
BO2_tempmax_ss	Maximum sea surface temperature
BO2_tempmean_ss	Mean sea surface temperature
BO2_tempmin_ss	Minimum sea surface temperature
BO2_temprange_ss	Range of sea surface temperature
BO2_curvelmax_ss	Maximum surface current velocity

BO2_curvelmean_ss	Mean surface current velocity
BO2_curvelmin_ss	Minimum surface current velocity
BO2_curvelrange_ss	Range of surface current velocity
BO2_dissoxmean_ss	Mean dissolved oxygen concentration
BO2_phosphatemean_ss	Mean mole concentration of phosphate at the sea surface
BO2_nitratemean_ss	Mean mole concentration of nitrate at the sea surface
BO2_ppmean_ss	Mean sea surface net primary productivity of carbon
BO2_salinitymean_ss	Mean sea surface salinity

Table D. Pearson's correlation **results** *among 29 environmental predictors*.

	1	2	3	4	5	6	7	8	9	10	11	12	13	14	15
1													*		
2	0.32														
3	-0.68	-0.11													
4	-0.47	-0.25	-0.23												
5	-0.64	-0.39	0.04	0.95											
6	0.34	0.02	0.41	-0.82	-0.68					*					
7	-0.77	-0.38	0.90	0.11	0.40	0.23			*						
8	0.59	0.13	0.15	-0.85	-0.79	0.96	-0.05			*					
9	-0.74	-0.44	0.88	0.10	0.40	0.26	0.99	-0.01							
10	0.46	0.04	0.28	-0.77	-0.66	0.98	0.13	0.98	0.17						
11	-0.44	-0.23	0.94	-0.45	-0.17	0.67	0.83	0.43	0.83	0.56					
12	0.92	0.43	-0.53	-0.68	-0.85	0.38	-0.78	0.61	-0.76	0.44	-0.33			*	
13	0.98	0.38	-0.69	-0.41	-0.61	0.32	-0.79	0.57	-0.76	0.45	-0.47	0.88			
14	-0.93	-0.43	0.64	0.58	0.78	-0.27	0.85	-0.52	0.83	-0.35	0.45	-0.99	-0.90		
15	-0.75	-0.18	0.89	-0.20	0.01	0.25	0.74	-0.01	0.70	0.08	0.80	-0.52	-0.73	0.61	
16	-0.95	-0.40	0.52	0.69	0.85	-0.46	0.74	-0.68	0.72	-0.53	0.29	-0.99	-0.91	0.98	0.54
17	-0.56	-0.14	0.16	0.36	0.40	-0.64	0.20	-0.72	0.17	-0.73	-0.06	-0.40	-0.66	0.36	0.21
18	0.72	0.46	-0.66	-0.44	-0.68	-0.04	-0.91	0.19	-0.91	0.00	-0.57	0.88	0.69	-0.91	-0.48
19	-0.42	0.11	0.08	0.06	-0.01	-0.40	-0.11	-0.45	-0.17	-0.51	-0.09	-0.15	-0.35	0.15	0.50
20	-0.93	-0.42	0.57	0.65	0.83	-0.37	0.79	-0.61	0.77	-0.44	0.36	-1.00	-0.90	0.99	0.55
21	-0.63	-0.31	0.97	-0.21	0.08	0.49	0.94	0.22	0.94	0.37	0.97	-0.55	-0.66	0.66	0.83
22	0.95	0.42	-0.50	-0.69	-0.83	0.46	-0.71	0.68	-0.69	0.54	-0.27	0.98	0.91	-0.97	-0.56
23	-0.71	-0.30	0.55	-0.07	0.05	-0.08	0.43	-0.28	0.41	-0.26	0.43	-0.41	-0.72	0.46	0.83
24	0.68	0.40	-0.87	-0.06	-0.34	-0.36	-0.96	-0.10	-0.96	-0.28	-0.85	0.72	0.66	-0.80	-0.73
25	-0.82	-0.23	0.25	0.50	0.53	-0.67	0.29	-0.82	0.25	-0.79	-0.01	-0.64	-0.81	0.61	0.52
26	-0.41	-0.10	-0.37	0.86	0.74	-0.99	-0.16	-0.97	-0.19	-0.98	-0.63	-0.46	-0.38	0.35	-0.19
27	-0.80	-0.23	0.17	0.63	0.65	-0.78	0.27	-0.90	0.23	-0.87	-0.11	-0.68	-0.80	0.63	0.38
28	0.86	0.35	-0.22	-0.84	-0.90	0.73	-0.46	0.88	-0.43	0.78	0.05	0.90	0.81	-0.85	-0.33
29	0.39	0.02	0.29	-0.90	-0.81	0.95	0.04	0.93	0.07	0.91	0.55	0.51	0.38	-0.40	0.27

	16	17	18	19	20	21	22	23	24	25	26	27	28	29
1														
2														
3														
4														
5														
6											*			
7														
8														
9														
10											*			
11														
12	*				*		*							
13														
14	*				*									
15														
16					*		*							
17	0.49													
18	-0.82	-0.07												
19	0.24	0.33	0.24											
20	0.99	0.42	-0.87	0.17			*							
21	0.52	0.06	-0.74	-0.06	0.58									
22	-0.99	-0.47	0.80	-0.30	-0.99	-0.50								
23	0.48	0.47	-0.18	0.73	0.44	0.50	-0.53							
24	-0.67	0.05	0.91	0.12	-0.73	-0.94	0.65	-0.37						
25	0.73	0.76	-0.23	0.74	0.66	0.16	-0.76	0.78	-0.16			*		
26	0.53	0.60	-0.05	0.42	0.45	-0.43	-0.54	0.15	0.27	0.71				
27	0.76	0.81	-0.27	0.62	0.69	0.09	-0.78	0.65	-0.12	0.98	0.81			
28	-0.93	-0.58	0.60	-0.39	-0.90	-0.20	0.94	-0.44	0.37	-0.83	-0.79	-0.89		
29	-0.56	-0.63	0.18	-0.14	-0.50	0.35	0.54	0.07	-0.19	-0.57	-0.93	-0.71	0.76	

Correlation coefficient values (lower left) are shaded blue-white-red for negative-zero-positive. Significant ($p < 0.05$) Pearson's test results are denoted with an asterisk (upper right). Column and row numbers refer to a specific environmental variable, noted in the key below. Table crosses two pages.

Key

	Variable Name
1	BO2_salinitymean_ss
2	BO2_ppmean_ss
3	BO2_nitratemean_ss
4	BO2_phosphatemean_ss
5	BO2_dissoxmean_ss
6	BO2_salinitymean_bdmean
7	BO2_ppmean_bdmean
8	BO2_nitratemean_bdmean
9	BO2_phosphatemean_bdmean
10	BO2_dissoxmean_bdmean
11	BO_bathymean

12 BO_ph

13 BO_calcite

14 BO2_curvelmax_ss

15 BO2_curvelmin_ss

16 BO2_curvelmean_ss

17 BO2_curvelrange_ss

18 BO2_curvelmax_bdmean

19 BO2_curvelmin_bdmean

20 BO2_curvelrange_bdmean

21 BO2_curvelmean_bdmean

22 BO2_tempmax_ss

23 BO2_tempmean_ss

24 BO2_tempmin_ss

25 BO2_temprange_ss

26 BO2_tempmax_bdmean

27 BO2_tempmean_bdmean

28 BO2_tempmin_bdmean

29 BO2_temprange_bdmean

Table E. All environmental predictor loadings on retained PCs.

Environmental Predictor	*PC1*	*PC2*	*PC3*
BO_bathymean	0.077	-0.321	0.041
BO_calcite	-0.081	-0.014	-0.035
BO_ph	0.047	-0.047	-0.398
BO2_curvelmax_bdmean	0.286	0.055	-0.100
BO2_curvelmax_ss	0.212	0.030	-0.234
BO2_curvelmean_bdmean	0.168	-0.076	-0.010
BO2_curvelmean_ss	0.186	0.099	-0.239
BO2_curvelmin_bdmean	0.238	-0.070	-0.155
BO2_curvelmin_ss	0.280	0.109	-0.141
BO2_curvelrange_bdmean	-0.203	0.027	0.056
BO2_curvelrange_ss	0.251	0.026	-0.177
BO2_dissoxmean_bdmean	-0.067	-0.313	-0.073
BO2_dissoxmean_ss	-0.287	-0.141	0.063
BO2_nitratemean_bdmean	-0.054	0.345	-0.044
BO2_nitratemean_ss	-0.021	0.018	-0.281
BO2_phosphatemean_bdmean	-0.042	0.338	0.122
BO2_phosphatemean_ss	-0.004	0.096	0.307

BO2_ppmean_bdmean	0.057	-0.285	0.233
BO2_ppmean_ss	0.180	-0.088	0.144
BO2_salinitymean_bdmean	-0.048	0.334	-0.092
BO2_salinitymean_ss	-0.006	0.014	-0.390
BO2_tempmax_bdmean	0.198	-0.244	0.114
BO2_tempmax_ss	0.251	-0.190	-0.064
BO2_tempmean_bdmean	0.283	-0.058	0.182
BO2_tempmean_ss	0.285	0.054	0.157
BO2_tempmin_bdmean	0.265	0.139	0.167
BO2_tempmin_ss	0.268	0.099	0.193
BO2_temprange_bdmean	-0.016	-0.338	-0.020
BO2_temprange_ss	-0.141	-0.224	-0.251

Table F. Sea surface predictor loadings for retained PCs.

Environmental Predictor	*PC1*	*PC2*
BO_calcite	-0.086	0.059
BO_ph	0.186	0.405
BO2_curvelmax_ss	0.328	0.156
BO2_curvelmean_ss	0.326	0.144

BO2_curvelmin_ss	0.395	-0.006
BO2_curvelrange_ss	0.375	0.062
BO2_dissoxmean_ss	-0.385	0.098
BO2_nitratemean_ss	0.096	0.283
BO2_phosphatemean_ss	-0.078	-0.351
BO2_ppmean_ss	0.154	-0.216
BO2_salinitymean_ss	0.131	0.401
BO2_tempmax_ss	0.276	0.004
BO2_tempmean_ss	0.285	-0.307
BO2_tempmin_ss	0.265	-0.348
BO2_temprange_ss	-0.122	0.384

Table G. Bottom depth predictor loadings for retained PCs.

Environmental Predictor	*PC1*	*PC2*
BO2_curvelmax_bdmean	0.058	-0.458
BO2_curvelmean_bdmean	0.147	-0.140
BO2_curvelmin_bdmean	0.169	-0.279
BO2_curvelrange_bdmean	-0.115	0.321
BO2_dissoxmean_bdmean	0.301	0.289

BO2_nitratemean_bdmean	-0.395	-0.097
BO2_phosphatemean_bdmean	-0.364	-0.127
BO2_ppmean_bdmean	0.343	0.029
BO2_salinitymean_bdmean	-0.387	-0.088
BO2_tempmax_bdmean	0.354	-0.197
BO2_tempmean_bdmean	0.205	-0.413
BO2_tempmin_bdmean	-0.014	-0.480
BO2_temprange_bdmean	0.345	0.182

Table H. Current velocity and temperature predictor loadings for retained PCs.

Environmental Predictor	*PC1*	*PC2*	*PC3*
BO2_curvelmax_bdmean	0.317	-0.066	0.098
BO2_curvelmax_ss	0.236	-0.257	0.268
BO2_curvelmean_bdmean	0.170	-0.044	0.054
BO2_curvelmean_ss	0.213	-0.118	0.454
BO2_curvelmin_bdmean	0.259	-0.167	0.116
BO2_curvelmin_ss	0.312	0.003	0.243
BO2_curvelrange_bdmean	-0.216	0.189	0.105
BO2_curvelrange_ss	0.272	-0.103	0.268

BO2_tempmax_bdmean	0.194	-0.251	-0.460
BO2_tempmax_ss	0.259	-0.299	-0.152
BO2_tempmean_bdmean	0.297	0.064	-0.338
BO2_tempmean_ss	0.308	0.202	-0.174
BO2_tempmin_bdmean	0.291	0.304	-0.091
BO2_tempmin_ss	0.291	0.286	-0.164
BO2_temprange_bdmean	-0.040	-0.471	-0.365
BO2_temprange_ss	-0.161	-0.496	0.088

Table I. Loci retained at each filtering step.

Filtering step	Loci
Total from *dDocent*	6738423
After removing indels	3987180
After maximum missing data per locus = 30%	94543
After minimum MAF = 0.05	9178
After minimum quality score = 20	9133
After removing SNPs out of Hardy Weinberg Equilibrium	5042
After retaining one SNP per RAD locus, with highest MAF	2075

All loci are SNPs after removing indels. MAF = minor allele frequency.

Table J. Pairwise F_{ST} *with 95% confidence intervals and genic differentiation test results*.

	CB_AK	YB_AK	AB_AK	BB_BC	SK_WA	JI_WA	KP_WA	EI_WA	CH_OR
CB_AK	-				*	*	*	*	*
YB_AK	**0.004** (0.001-0.005)	-		*	*	*	*	*	*
AB_AK	**0.004** (0.001-0.007)	**0.004** (0.001-0.007)	-						
BB_BC	**0.008** (0.005-0.011)	**0.005** (0.003-0.007)	**0.005** (0.001-0.008)	-	*	*	*	*	*
SK_WA	**0.011** (0.009-0.013)	**0.009** (0007-0.011)	**0.008** (0.004-0.011)	**0.006** (0.004-0.009)	-		*	*	*
JI_WA	**0.015** (0.012-0.018)	**0.012** (0.010-0.014)	**0.012** (0.008-0.017)	**0.007** (0.006-0.010)	**0.002** (0.001-0.004)	-	*	*	*
KP_WA	**0.010** (0.009-0.014)	**0.009** (0.007-0.012)	**0.008** (0.003-0.012)	**0.007** (0.005-0.010)	**0.004** (0.002-0.006)	**0.006** (0.005-0.010)	-		*
EI_WA	**0.011** (0.009-0.014)	**0.008** (0.007-0.012)	**0.011** (0.007-0.016)	**0.006** (0.005-0.010)	**0.005** (0.003-0.006)	**0.005** (0.004-0.006)	**0.001** (0.000-0.003)	-	*
CH_OR	**0.009** (0.006-0.011)	**0.008** (0.006-0.011)	**0.007** (0.003-0.010)	**0.009** (0.007-0.013)	**0.008** (0.007-0.010)	**0.010** (0.008-0.013)	**0.004** (0.001-0.005)	**0.005** (0.004-0.008)	-

Pairwise F_{ST} *(and 95% confidence intervals) in the lower left and pairwise genic differentiation test results (*:* $p < 0.05$*) in the upper right of the table. Confidence intervals were generated by bootstrapping across SNPs with 100 repetitions. Cells with higher* F_{ST} *values are shaded darker green. Site abbreviations provided in Table 1.*

***Table K. Number of SNPs with at least strong evidence for correlation with environmental variables in* A. californicus.**

Environmental predictor	*Depth*	S_C	S_P	*Biological processes*
Mean salinity	S	38	4	signal transduction (0.259),<br>cell organization and biogenesis (0.207),<br>protein metabolism (0.207),<br>cell cycle and proliferation (0.138),<br>RNA metabolism (0.086),<br>transport (0.069),<br>DNA metabolism (0.034)
Mean nitrate	S	34	4	signal transduction (0.294),<br>cell organization and biogenesis (0.235),<br>developmental processes (0.216),<br>cell cycle and proliferation (0.078),<br>transport (0.078),<br>protein metabolism (0.059),<br>DNA metabolism (0.039)
Temperature range	S	31	2	cell organization and biogenesis (0.387),<br>signal transduction (0.29),<br>RNA metabolism (0.097),<br>developmental processes (0.065),<br>DNA metabolism (0.065),

				protein metabolism (0.065),<br>stress response (0.032)
pH	S	30	3	cell organization and biogenesis (0.353),<br>developmental processes (0.324),<br>signal transduction (0.265),<br>DNA metabolism (0.059)
Mean temperature	B	27	1	signal transduction (0.5),<br>transport (0.5)
Mean phosphate	S	26	2	cell organization and biogenesis (0.522),<br>signal transduction (0.391),<br>DNA metabolism (0.087)
Minimum temperature	S	24	1	signal transduction (0.5),<br>transport (0.5)
Minimum temperature	B	22	1	signal transduction (0.5),<br>transport (0.5)
Mean primary productivity	S	19	2	cell organization and biogenesis (0.308),<br>protein metabolism (0.231),<br>signal transduction (0.231),<br>RNA metabolism (0.128),<br>cell cycle and proliferation (0.103)
Mean temperature	S	18	1	signal transduction (0.5),<br>transport (0.5)
Mean current velocity	B	14	2	cell organization and biogenesis (0.367),

				transport (0.245),<br>developmental processes (0.204),<br>signal transduction (0.184)
Mean dissolved oxygen	S	14	1	developmental processes (0.522),<br>transport (0.217),<br>signal transduction (0.174),<br>RNA metabolism (0.087)
Maximum temperature	S	14	2	stress response (1)
Maximum current velocity	B	13	1	transport (0.333),<br>developmental processes (0.167),<br>stress response (0.148),<br>protein metabolism (0.13),<br>signal transduction (0.093),<br>cell organization and biogenesis (0.037),<br>death (0.037),<br>RNA metabolism (0.037),<br>cell cycle and proliferation (0.019)
Minimum current velocity	B	12	NA	NA
Minimum current velocity	S	12	1	developmental processes (0.522),<br>transport (0.217),<br>signal transduction (0.174),<br>RNA metabolism (0.087)

Range in current velocity	S	12	1	DNA metabolism (1)
Maximum temperature	B	12	NA	NA
Mean primary productivity	B	11	1	DNA metabolism (1)
Mean dissolved oxygen	B	10	1	transport (0.42),<br>cell organization and biogenesis (0.174),<br>developmental processes (0.174),<br>signal transduction (0.072),<br>protein metabolism (0.058),<br>cell adhesion (0.043),<br>cell cycle and proliferation (0.043),<br>RNA metabolism (0.014)
Range in current velocity	B	9	1	cell organization and biogenesis (0.571),<br>signal transduction (0.429)
Mean phosphate	B	9	1	signal transduction (0.326),<br>stress response (0.256),<br>developmental processes (0.116),<br>protein metabolism (0.093),<br>transport (0.093),<br>death (0.07),<br>cell-cell signaling (0.023),<br>cell cycle and proliferation (0.023)

Maximum current velocity	S	8	1	protein metabolism (1)
Mean current velocity	S	8	1	protein metabolism (1)
Mean nitrate	B	6	NA	NA
Calcite	S	5	NA	NA
Mean salinity	B	5	NA	NA
Range in temperature	B	5	NA	NA
Mean bathymetry	B	3	NA	NA

Column Depth *refers to whether the variable was measured at sea surface (S) or mean bottom depth (B). Column* N_C *refers to the number of correlated SNPs. Column* N_B *refers to the number of correlated SNPs with associated gene ontology slim terms for biological processes.*

Table L. Loadings for RDA using sea surface predictors.

	RDA1	RDA2
PC1	0.266	-0.964
PC2	-0.964	-0.266

The loadings for each PC are presented in S5 Table.

Table M. Loadings for RDA using current and temperature predictors.

	RDA1	RDA2	RDA3
PC1	0.456	-0.742	0.492
PC2	0.810	0.574	0.116
PC3	-0.368	0.346	0.863

The loadings for each PC are presented in S8 Table.

Table N. Counts of gene ontology slim terms in the database.

Gene ontology slim term	*n*
other metabolic processes	4102
other biological processes	3320
developmental processes	3092
cell organization and biogenesis	1712
transport	1398
protein metabolism	1261
stress response	1000
signal transduction	917
cell cycle and proliferation	675
RNA metabolism	574
cell-cell signaling	302
DNA metabolism	299
death	267
cell adhesion	107

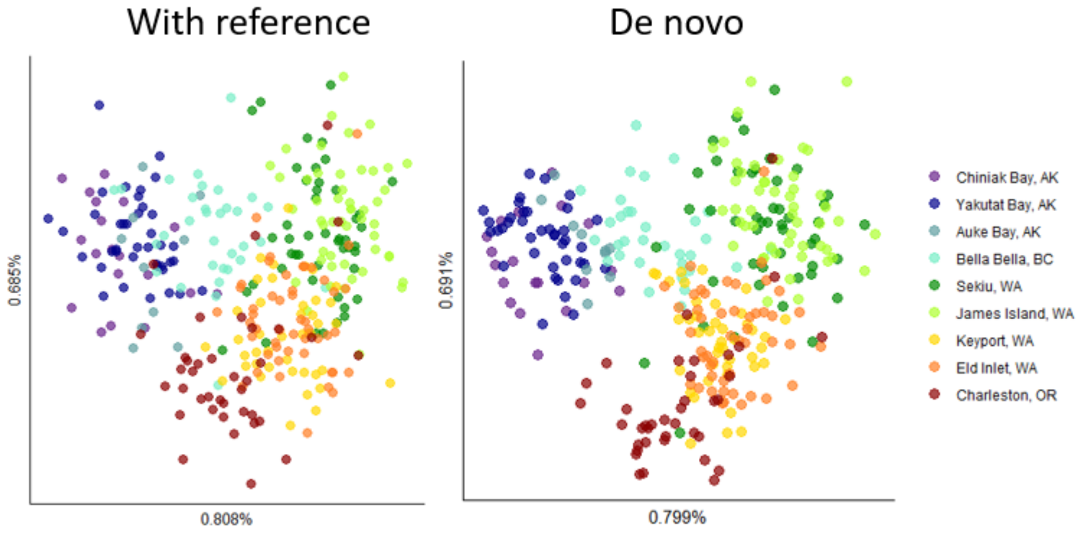

***Fig A. Individuals plotted across first two principal components from principal component analysis, demonstrating similarity between the assembly with reference to the* de novo *assembly*.**

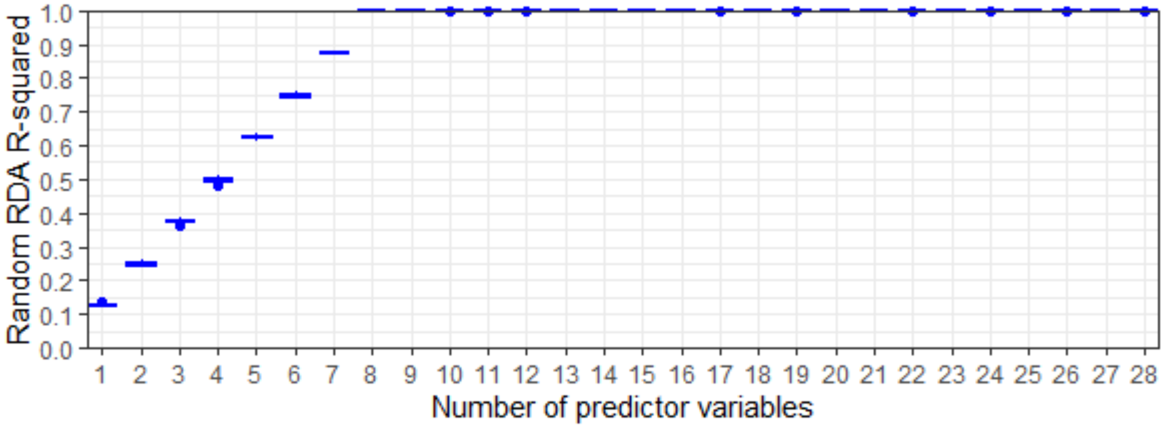

Fig B. Overfitting of RDA models occurs quickly with the addition of predictor variables. *Using randomly generated data based on the mean and standard deviations of environmental predictor data used in this study, we demonstrated that R-squared reaches 1 with the addition of 8 predictor variables.*

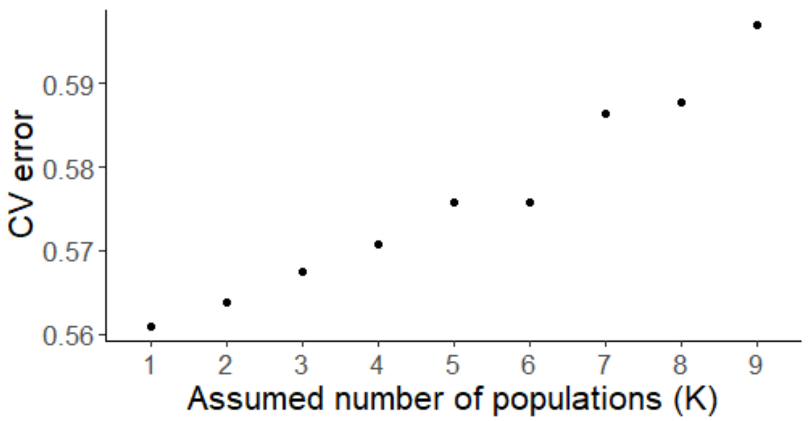

Fig C. Cross validation error by number of assumed underlying populations in ADMIXTURE clustering analysis.

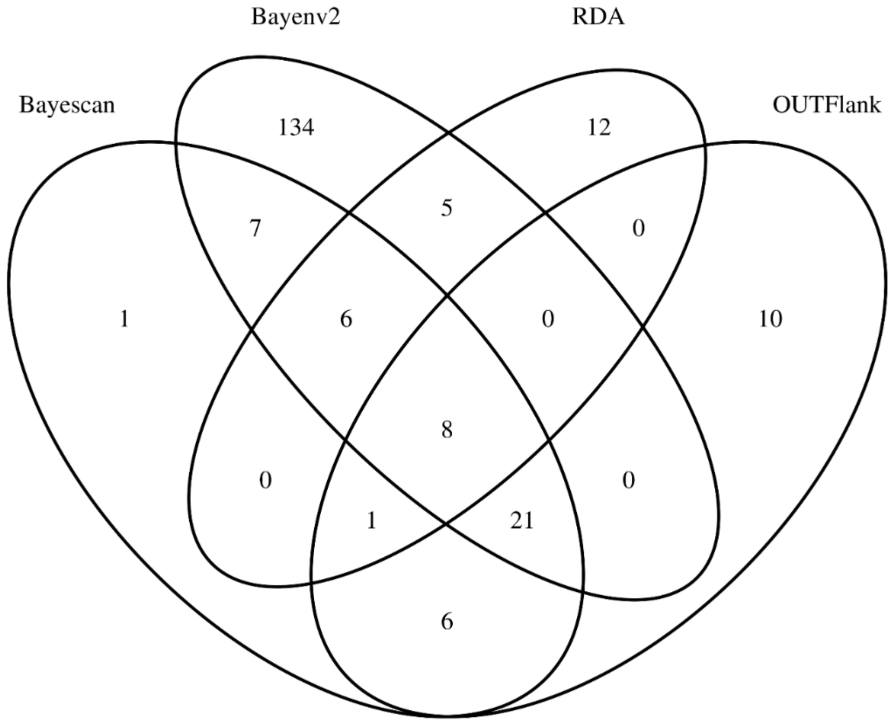

Fig D. Venn diagram showing overlap among putatively adaptive SNPs by method.
